# Green Synthesis of Highly Monodisperse and Spherical Ag Nanoparticles by a Combination of *Teucrium ramosissimum* Desf. (Lamiaceae) Extracts with Emphasis on the Stabilizing and Capping Biomolecules

**DOI:** 10.1021/acssuschemeng.3c07504

**Published:** 2024-02-27

**Authors:** Rim Bouhajeb, Ana C. Abreu, Silvia Fernández, Mohamed Bayrem-Ghedira, Leila Chekir-Ghedira, Ignacio Fernández, Rafael Contreras-Caceres

**Affiliations:** † Unit of Bioactive and Natural Substances and Biotechnology UR17ES49, Faculty of Dental Medicine and Faculty of Pharmacy, 474418 University of Monastir, Monastir 5000, Tunisia; ‡ Department of Chemistry and Physics, Research Centre CIAIMBITAL, 16721 University of Almería, Almería 04120, Spain

**Keywords:** Teucrium ramosissimum Desf, green chemistry, silver nanoparticles, NMR metabolomics

## Abstract

Here, the aqueous (T_aquo_) and hydroethanolic (T_hydro_) extracts of the *Teucrium ramosissimum* Desf. are used for the synthesis of one of the most spherical and monodisperse Ag nanoparticles (Ag NPs) reported by green chemistry. Several parameters, such as pH, amount of extract, reaction time, and reaction temperature, are investigated. The optimized Ag NPs are obtained at a pH value of ca. 10, using 1 mL of extract, a reaction time of 4 h, at 60 °C, and allowing an incubation period at room temperature. The average particle size ranged between 18 and 22 nm in all cases, and no significant differences were observed between the T_aquo_ or the T_hydro_ extracts. Apart from that, the principal bioactive molecules responsible for the reduction process were identified by nuclear magnetic resonance spectroscopy, and the molecules incorporated on the Ag surface were determined by headspace-solid phase microextraction (HS-SPME), followed by gas chromatography/quadrupole-mass spectrometry (GC–qMS). In both cases, we found that phenolic acids, phenylpropanoids, citric acid, and malic acid are molecules involved in the reduction process, and some of them are found on the Ag NPs surface in their oxidized form. Moreover, a stabilization study as a function of pH is also presented that confirms the high stability of our fabricated Ag NPs. UV–vis analysis confirmed the presence of the Ag plasmon band as well as the particle stability, and transmission electron microscopy images demonstrate that our synthesized Ag NPs are some of the most spherical and monodisperse biobased Ag nanosystems presented in the literature. Ag NPs were also analyzed by scanning electron microscopy, energy-dispersive X-ray spectroscopy, selected area electron diffraction, and Fourier-transform infrared spectroscopy.

## Graphical abstract

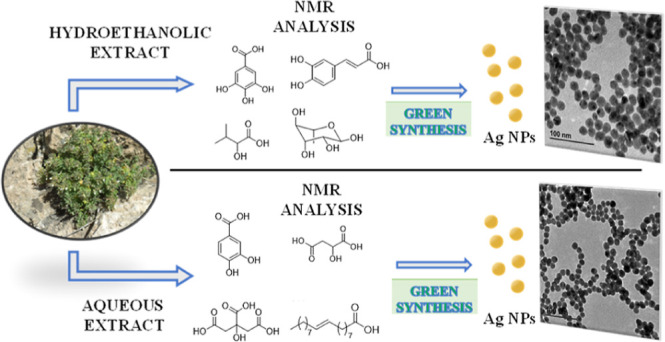

## 1 Introduction

During the last decades, nanotechnology has become an emerging and promising research field involving the synthesis and application of materials at the nanoscale level.^1^ Nanoparticles (NPs) exhibit different and improved properties compared to their bulk counterparts.^2–5^ Specifically, colloidal dispersion containing noble metal NPs based on Au, Ag, and Cu possess unique properties,^6–8^ that make them excellent candidates to be used in sensing,^9^ catalysis,^10^ or materials for diagnosis and therapy.^11–14^ In this context, the synthesis of silver nanoparticles (Ag NPs) has become a promising research area in colloidal chemistry due to several reasons: (i) Ag possesses the strongest surface plasmon band,^15^ thus making Ag NP the best candidates for sensing application,^16^ (ii) Ag NPs have demonstrated high capability in catalytic applications,^17,18^ and (iii) Ag NPs have been extensively reported as colloidal systems with remarkable antibacterial properties.^19,20^ These antibacterial activities have been demonstrated against numerous Gram-positive and Gram-negative bacteria.^21–23^ The literature presents various mechanisms, observed either in combination or independently, by which Ag NPs manifest their antibacterial properties:^24–26^ (i) destabilization and damage to the inner membrane;^27,28^ (ii) interaction within the cell,^29^ and (iii) the release of silver ions from nanoparticles,.^30–32^ For this reason, extensive research reports have delineated the efficacy of Ag NPs against diverse bacteria such as *E. coli*, *Pseudomonas aeruginosa*, and *Staphylococcus aureus*, among others.^33^ Related to the physicochemical properties affecting the antibacterial activity, several reports have already addressed this issue, revealing that positively charged,^34^ and reduced sizes are usually the most active.^35–37^ We are currently testing our biosynthesized Ag NPs both independently and when embedded within chitosan-free and chitosan-containing nanofibers. Therefore, for all these reasons, numerous methodologies have been developed focused on the synthesis of Ag NPs.^38–40^ These procedures are defined as “traditional methods” and are based on the chemical reduction of a silver salt in the presence of a reducing agent. For example, the Tollens synthesis method provides Ag NPs by using Ag­(NH_3_)_2_^+^ as a metal precursor, employing an aldehyde as the reducing agent.^41,42^ However, silver nitrate (AgNO_3_) is the most commonly used metal source to obtain Ag NPs, and sodium borohydride (NaBH_4_) or sodium citrate are used as reducing agents.^37,39,43,44^ Unfortunately, these approaches have some inherent problems, the obtained Ag NPs present high polydispersity, and they incorporate toxic compounds on the surface of Ag NPs, such as NaBH_4_, which reduce the potential application for in vivo investigations. For this reason, in recent years, the synthesis of Ag NPs has been carried out using safer, healthier, and more environmentally friendly methods arising from vegetal origin.^8,45,46^ These procedures are encompassed within the designated green chemistry strategies and are a consequence of the fact that the bioactive compounds contained in these vegetal extracts serve the dual functions of both reducing and capping agents. In this sense, plant, lichen, or fruit extracts, as well as enzymes or bacteria, are currently used in green chemistry procedures to obtain Ag NPs.^38,45,47–51^ These extracts include a multitude of natural compounds, such as polysaccharides, antioxidants, flavonoids, terpenoids, phenolic compounds, and organic acids,^45,52,53^ which makes plant extracts highly bioactive and amenable to green chemistry synthesis.^45,52,54^ As an example, Ag NPs have been synthesized using tea leaf, coffee extracts, grape pomace, beet juice, and also with extracts derived from pepper, blueberry, geranium, aloe vera, and hibiscus, among others.^55–62^ Table S1 includes previously reported protocols for the green synthesis of Ag NPs performed as a function of several parameters, such as pH,^63–65^ temperature,^19,60^ reaction time,^50,55^ or incubation time.^19,66^ This table includes the type of extract used for the synthesis of Ag NPs, the parameters investigated in each case, the particle size, and the reference. Indeed, this table provides empirical evidence demonstrating that the utilization of these vegetal extracts leads to the formation of polydisperse particle distributions, wherein deviations from the average diameter typically fall within the range of several to tens of nanometers.

*Teucrium ramosissimum* Defs. (a Lamiaceae plant family from the south of Tunisia) has been traditionally applied in medicine due to its therapeutic properties, including wound healing,^67,68^ anti-inflammatory,^69^ antimicrobial,^70^ analgesic,^71^ and antioxidant.^72^ Recently, the extract of this plant has been incorporated into electrospun polylactic acid nanofibers (PCL NFs), providing interesting drug-release properties in the wound-healing process in induced pressure ulcers in mice.^73^ The presence of specific flavonoids, polyphenols, and sesquiterpenes, among other organic molecules, in the ethanolic extract of *T. ramosissimum* is an important motivation for its use in green chemistry protocols.

Within this framework and given the abundance of molecules present in a plant extract, it is valuable to ascertain the precise molecule or molecules responsible for the reduction process and, consequently, involved in the generation of Ag NP. In this vein, we have recently identified the specific molecules responsible for the reduction process from Ag^+^ to Ag^0^ in the presence of the ethanolic extract of a specific blueberry family (*V. corymbosum* L.).^45^ In this work and through NMR investigations, we were able to prove that the caffeoylquinic derivatives quercetin, gallic acid, citric acid, and malic acid are molecules involved in the generation of Ag NPs. However, the monodispersity of the synthesized NPs could be improved. It is important to note that

Here, we present the green synthesis of Ag NPs using, for the first time, the aquo and the hydroethanolic extract of the *Teucrium ramosissimum* Desf. extracts (T_aquo_ and T_hydro_),^74^ showing remarkable efficiency in terms of highly spherical morphology and monodispersity. To do that, several parameters have been investigated, such as the pH value, the reaction time, the reaction temperature, the concentration of extract, and the incorporation of an incubation step. Two distinct types of solvent systems were explored for the green synthesis, considering that the extracted compounds typically differ. This choice stems from the fact that the compounds extracted are often different due to solvent factors such as polarity, hydrophobicity, hydrophilicity, and boiling point, and they can influence Ag NP quality. As was previously mentioned, the Supporting Information provides a detailed table (Table S1) containing an exhaustive bibliographic screening of published work concerning the green synthesis of Ag NPs. It is important to mention that all these protocols of synthesis generate Ag NPs with higher polydispersity compared with our protocol, and in several cases, no spherical shape was obtained. As an added value compared with all these reported works, herein we are able to elucidate the main bioactive molecules responsible not only for the reduction of the metal precursor but also for the capping molecules attached to the metal surface that provide ulterior stabilization to the biosynthesized Ag NPs. This colloidal stability was performed as a function of pH. Nuclear magnetic resonance (NMR) spectroscopy and headspace-solid phase microextraction (HS-SPME), followed by gas chromatography/quadrupole-mass spectrometry (GC–qMS) helped us in the unraveling of all these chemical species. The stability study as a function of pH was conducted by UV–vis spectroscopy and demonstrated the higher stability of the biosynthesized Ag NPs compared with those obtained using NaBH_4_.^39,44^

## 2 Materials and Methods

### 2.1 Materials

Silver nitrate (99%), sodium hydroxide, and absolute ethanol were purchased from Merck and used without further purification. Deuterium oxide and 3-(trimethylsilyl) propionic-2,2,3,3-*d*_4_ acid (TSP) were purchased from Eurisotop (Saint-Aubin, France). Water was purified by using a Milli-Q system (Millipore).

### 2.2 Characterization

UV–vis measurements of aqueous colloidal solutions were recorded using a Jasco V-730 spectrophotometer with a quartz cuvette with a 1 cm length path. Transmission electron microscopy (TEM) images were acquired on a JEOL JEM 1400 operating at an acceleration voltage of 80 kV. Samples were prepared by drying a 10 μL drop of colloidal suspension on a carbon-coated copper grid. SEM–EDX was performed using a Hitachi S-3500N microscope working at 15 kV. Samples were previously sputtered with carbon to minimize the charging effect. SAED was acquired on a Talos F200X instrument at an acceleration voltage of 200 kV. FTIR spectra were recorded using a FT-R ALPHA spectrometer in attenuated total reflectance (ATR) mode. Approximately 1.0 mg of neat Ag NP particles were used in each case.

### 2.3 Collection of the Starting Plant Material

A voucher specimen (Tr/02/05) was deposited at the Herbarium of the Department of Pharmacognosy, Faculty of Pharmacy, University of Monastir, Tunisia. Aerial parts of *Teucrium ramosissimum* Desf. were collected in January 2019 from the mountainous region of Gafsa in southeast Tunisia. The botanical identification of plant material was approved by Professor Fathia Harzallah-Skhiri, a consolidated plant taxonomist (Institute of Biotechnology of Monastir, University of Monastir, Tunisia). The voucher specimen was deposited in the herbarium of the Laboratory of Pharmacognosy, Faculty of Pharmacy of Monastir, Tunisia.

### 2.4 Isolation of the Hydroethanolic Extract (T_hydro_) of *T. ramosissimum*

The aerial parts of *T. ramosissimum* were dried in the shade at room temperature and then preserved in paper bags to avoid possible fungal contamination. The plant materials were crushed to a fine powder. After that, it was macerated in absolute ethanol (1:10 w/v powder/ethanol, respectively) for 3 days under stirring. The infusion was filtered using filter paper (Whatman no. 1). The filtrate was concentrated under reduced pressure using rotary evaporation at 40 °C until complete withdrawal of the ethanol, followed by freezing at −20 °C and subsequent lyophilization. The powdered crude extract was stored at 0 °C until further use.

### 2.5 Isolation of the Aqueous Extract (T_aquo_) of *T. ramosissimum*

The aerial parts of *T. ramosissimum* were dried in the shade at room temperature and then preserved in paper bags to avoid possible fungal contamination. The plant materials were crushed to a fine powder. Subsequently, 50 g of powder was boiled in 500 mL (1:10 w/v powder/water, respectively) of distillated water for 20 min. The infusion was filtered using filter paper (Whatman no. 1). The filtrate was followed by lyophilization. The powdered crude extract was stored at 0 °C until further use.

### 2.6 Synthesis of Ag NPs

The synthesis of Ag NPs using the aqueous (T_aquo_) and hydroethanolic (T_hydro_) extracts of *T. ramosissimum* was carried out following a previous procedure reported by some of us, but with some modifications.^45^ First, the T_aquo_ and the T_hydro_ extracts to be used in the synthesis were prepared by dissolving 1 mg of each previously obtained powder in 1 mL of Milli-Q water or ethanol, respectively. After that, for a typical synthesis using the optimized conditions (see Section 3.1), 1 mL of each freshly prepared plant extract was added to 50 mL of AgNO_3_ (1 mM) under low magnetic stirring at 60 °C, and the mixture was maintained at these conditions for 30 min in darkness. Then, some drops of NaOH (0.5 M) were added to the mixture to reach a pH value of ca. 10.5, and the mixture was stirred for 4 h. After that, the colloidal dispersion was allowed to incubate overnight (12 h) at room temperature. Finally, the synthesized Ag NPs were centrifuged at 8000 rpm for 30 min, and the resulting pellet diluted was in 10 mL of Milli-Q water.

Variable modifications such as pH values of 7.2, 8.8, and 9.5 for T_aquo_ and 6.75, 8.8, and 9.3 for T_hydro_, using both 1 and 2.5 mL of extracts, reaction times of 6 h with and without overnight incubation, and synthesis performed at 80 °C were also assayed. Section 3.1 includes the characterization of each of the described synthesis.

### 2.7 NMR Measurements

^1^H NMR measurements were performed as the analytical platform chosen to determine the structure of those metabolites present in both aqueous and hydroethanolic extracts and to identify those specifically involved in the generation of Ag NPs. After the synthesis of the NPs, the colloidal suspension was precipitated and separated by centrifugation (15,000 rpm for 30 min). The blanks were prepared by mixing 50 mL of Milli-Q water with 1 mL of freshly prepared plant extract in each case, but without silver nitrate. All supernatants were freeze-dried for 72 h. Then, 0.6 mL of D_2_O phosphate buffer (pH 10) containing TSP at 0.1 mg/mL, which was used as an internal standard, was added to each sample. The mixtures were sonicated for 20 min, vortexed for 5 min at 600 rpm, and centrifuged for 5 min at 13,500 rpm. Three independent experiments were performed to obtain several replicates for multivariate data analysis. The supernatants (500 μL) were transferred to oven-dried 5 mm NMR tubes for NMR analysis. A Bruker Avance III 600 spectrometer operating at a proton frequency of 600 MHz with a 5 mm QCI quadrupole resonance pulse field gradient cryoprobe and a thermostated NMR autosampler (SampleJet) with 480 positions were used. The acquisition and processing of NMR spectra were performed using the TOPSPIN software (version 3.6.2). All spectra were obtained in the absence of rotation at 293 ± 0.1 K using a NOESY presaturation pulse sequence (Bruker noesygppr1d pulse sequence). The acquisition parameters were: NS = 32, TD = 64 K, SW = 20.0 ppm, AQ = 2.73 s, D1 = 5 s, FID resolution = 0.37 Hz, D8 = 10 ms, and RG = 57. The spectra were automatically phased, baseline corrected, and referenced to the TSP signal, which was set to 0.0 ppm. For structure elucidation purposes, 2D NMR experiments were acquired using standard Bruker sequences, namely, ^1^H–^1^H correlation spectroscopy (COSY), ^1^H–^1^H total correlation spectroscopy (TOCSY), ^1^H–^13^C edited heteronuclear single quantum coherence (HSQC), and ^1^H–^13^C heteronuclear multiple bond coherence (HMBC).

### 2.8 Head Space Gas Chromatography/Quadrupole-Mass Spectrometry (HS-SPME-GC–qMS)

SPME fibers (Supelco, Bellefonte, PA, USA) based on divinylbenzene/carboxen/polydimethylsiloxane 50/30 um (DVB/CAR/PDMS) were assessed for sampling efficiency of the volatile components. Prior to headspace sampling, all SPME fibers were conditioned for 30 min at 40 °C. An Agilent Technologies 6980N Gas Chromatograph (GC) with a HP-5MS column and an Agilent 9575 Mass Spectrometer Detector (MSD) with MSD ChemStation Acquisition Software (E.02.02 SP1 Agilent) were used for all analyses. Further details can be found in reference 45.

## 3 Results and Discussion

### 3.1 Green Synthesis of Ag NPs Using the Plant Extracts of *T. ramosissimum*

#### 3.1.1 Effect of the pH

As is well-known, pH provides an important effect on the redox potential of the biomolecules present in the plant extracts, which are responsible for the reduction process during the fabrication of Ag NPs, from Ag^+^ to Ag^0^.^19,45,59,65^ In this regard, the synthesis of Ag NPs was carried out at different pH values, which were obtained by adding different amounts of a 0.5 M NaOH solution to a mixture containing AgNO_3_ and the T_aquo_ or T_hydro_ extracts (see Materials and Methods for details). The synthesis was performed at 60 °C because this was the temperature used in a previously green synthesis method reported by some of us.^45^ Initially, the morphological characterization of the synthesized Ag NPs as a function of pH is included in Figure S1. This figure includes representative TEM images of Ag NPs obtained at the lowest pH values for the T_aquo_ and T_hydro_ extracts (see the experimental section for details). Upon observation, the TEM images show the presence of larger and irregular Ag NPs at pH values of 7.2 and 8.8 for T_aquo_ (Figure S1A,B), and 6.75 and 8.8 for T_hydro_ (Figure S1C,D), thus showing an undesirable high polydispersity in terms of particle size. Figure 1 includes representative TEM images of Ag NPs obtained at the highest pH values (included in the UV–vis analysis). Figure 1A,B include TEM images for Ag NPs obtained with the T_aquo_ extract at pH values of 9.5 and 10.3, respectively. For the T_hydro_ extract, Figure 1C,D show TEM images of Ag NPs obtained at pH 9.0 and 10.0, respectively. As can be observed, the presence of larger and irregular particles is considerably reduced, and instead of that, spherical and highly monodisperse Ag NPs were mainly obtained at these pH values. For this reason, the pH value used for the investigation of the other parameters was a pH value of ca. 10. The average particle size and the particle size distribution for Ag NPs obtained using the T_aquo_ extract at pH values of 9.5 and 10.3 are included in Figure S2A,B, respectively. These values were measured at 18.9 ± 3.7 and 18.2 ± 3 nm, respectively. Figure S2C,D include the particle size distribution and the average particle size for Ag NPs obtained using the T_hydro_ extract at pH 9.0 and 10.0, respectively, resulting in 20.8 ± 3.8 and 20.3 ± 3.7 nm.

**Figure 1:**
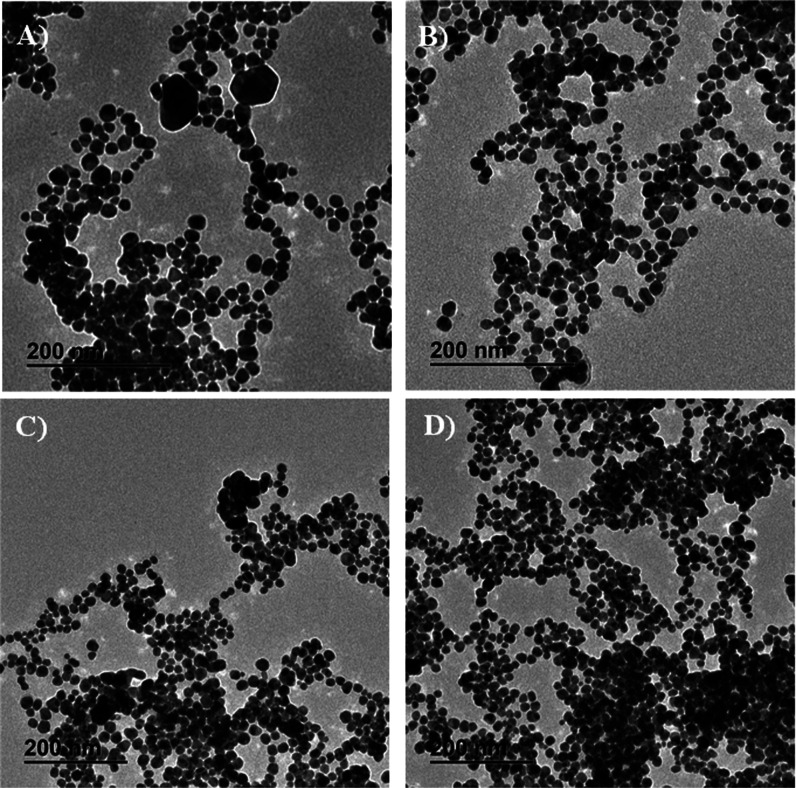
Representative TEM images for Ag NPs obtained at pH values of (A) pH 9.5 and (B) pH 10.3 using the T_aquo_ extract, and at pH values of (C) pH 9 and (D) pH 10 using the T_hydro_ extract.

Figure 2A,B show the normalized UV–vis spectra of the resulting Ag NP dispersions obtained at the two highest pH values using the T_aquo_ and T_hydro_ extracts, respectively. As shown, a maximum plasmon band is observed in all cases, suggesting the generation of Ag NPs. Indeed, the presence of a minimum at ∼320 nm also indicates the presence of Ag NPs, which is characteristic of the interband transition in the metal that damps the plasma oscillation in this spectral region.^75^ In both cases, only small differences were observed as a function of the pH. Initially, for the T_aquo_ synthesis, the maximum plasmon band was located at 411 nm when the reaction was performed at a pH value of 9.5 (Figure 2A, red line). As the pH value increased to 10.3, a displacement of the maximum plasmon band to 399 nm was produced (Figure 2A, black line). For the T_hydro_ synthesis, the maximum plasmon band position was located at 413 nm when Ag NPs were prepared at pH 9 (Figure 2B, red line), and again, it was shifted to 401 nm at a pH of 10 (Figure 2B, black line). This displacement suggests a decrease in the particle dimension because the plasmon band position depends on the particle size.^76^ Another difference observed in the UV–vis is a little narrower spectrum when the reaction is performed at lower pHs values, which is observed in both cases, thus suggesting more monodispersed Ag NPs at higher pHs values.

**Figure 2:**
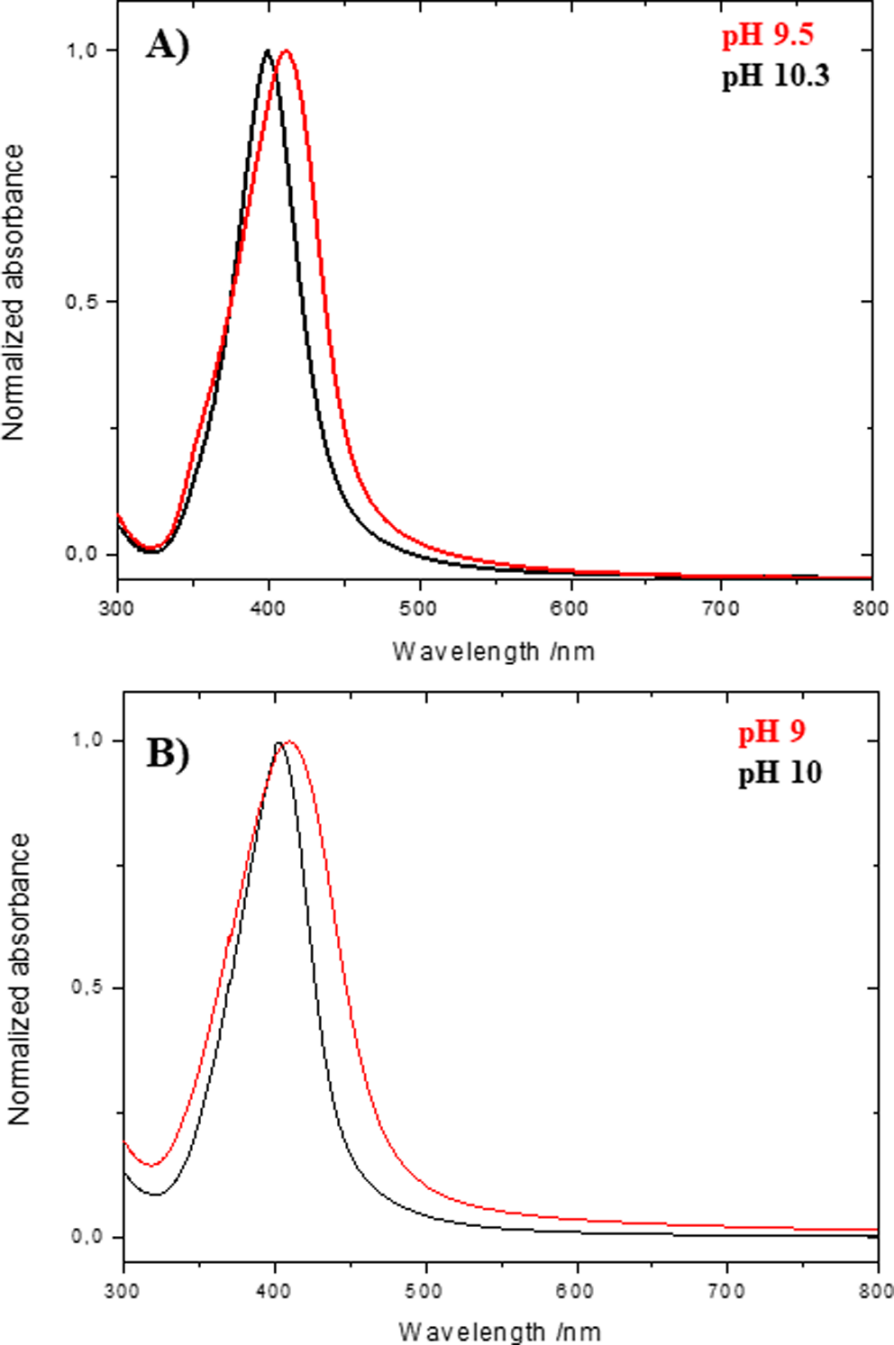
UV–vis spectra for Ag NP dispersions synthesized at different pH values using (A) T_aquo_ and (B) T_hydro_ extracts.

#### 3.1.2 Effect of the Concentration of Extracts

The amount of plan extract added to the AgNO_3_ solution is also a parameter frequently investigated when the synthesis of Ag NPs is performed by green chemistry strategies.^19,45,50,56,58^ When the amount of both T_aquo_ and T_hydro_ extract was increased from 1 to 2.5 mL (see experimental section for details), the presence of large irregular and nondefined Ag NPs could be observed at several pH values (6.8, 7.3, and 9.0). In fact, at the highest pH value assayed of 10.0, the number of large particles is reduced compared to the previous pH values, but still, the Ag NPs appear very aggregated, with the presence of two different particle size distributions. Figure S3 includes four representative TEM images of these Ag NPs synthesized by adding 2.5 mL of the T_aquo_ extract at different pH values. The situation is almost identical when the same amount of T_hydro_ extract is used during the synthesis of Ag NPs. Figure S4 shows representative TEM images of Ag NPs obtained by adding 2.5 mL of the T_hydro_ extract at different pH values (7.0, 8.5, 9.0, and 10.0). Again, the presence of irregular and large particles is observed in all cases, even at the highest pH value of 10.0. The increase in the amount of plant extract in both T_aqueo_ and T_hydro_ extracts resulted in the generation of Ag NPs with a considerably reduced quality, and consequently, the investigation of the rest of the parameters was performed by adding 1 mL of each extract.

#### 3.1.3 Effect of the Reaction Time with and without Incubation

The reaction time also plays an important role in the green synthesis of Ag NPs,^19,50,51,55,60^ and after optimizing the pH value and the amount of extract to obtain the best Ag NPs, the synthesis was performed at two different reaction times, 4 and 6 h, again based on our previous reported protocol.^45^ Indeed, after these periods of synthesis, we have investigated the possibility of performing on the just generated Ag NPs an incubation period of 12 h at room temperature. The term incubation denotes that the synthesis includes a step at room temperature in the dark without any stirring. The incubation time in the green synthesis of Ag NPs influences the reaction kinetics, nucleation and growth, stabilization, and overall characteristics of the nanoparticles. Optimization of this parameter is essential to tailoring the properties of the nanoparticles for specific applications. Figure 3 includes representative TEM images of Ag NP after 4 and 6 h of reaction without incubation using the T_aquo_ and T_hydro_ extracts. As observed, spherical Ag NPs were obtained for both reaction periods. For the T_aquo_ extract, the presence of larger and irregular morphologies is not observed at both reaction times (4 h, Figure 3A and 6 h, Figure 3B). A more detailed observation of the TEM images suggests that the Ag NPs synthesized during 4 h (Figure 3A) appear more regular compared with those obtained at 6 h of reaction (Figure 3B). The average particle size and the particle size distribution for Ag NPs obtained at 4 and 6 h of reaction without incubation and using the T_aquo_ extract are included in Figure S5A,B, respectively, resulting in 20.2 ± 2.9 and 20.8 ± 3.3 nm. Representative TEM images for Ag NPs obtained at 4 and 6 h of reaction using the T_hydro_ extract without incubation are included in Figure 3C,D, respectively. As observed, spherical Ag NPs were obtained at both reaction times with the absence of larger and nondefined nanoparticles. Figure S5C,D include the average particle size and the particle size distribution for Ag NPs synthesized at 4 and 6 h of reaction using the T_hydro_ extract without an incubation period. The average particle size was measured at 19.8 ± 3.1 and 20.2 ± 5.5 nm, respectively. As observed, only a small increase in the average particle size was produced when the reaction time was increased for 4 to 6 h in both extracts (T_aquo_ and T_hydro_). It is important to note that only slightly differences were observed between the synthesis performed at 4 and 6 h. However, based on the lower standard deviation obtained at 4 h of reaction, we consider that this period of time is more convenient for green chemistry.

**Figure 3:**
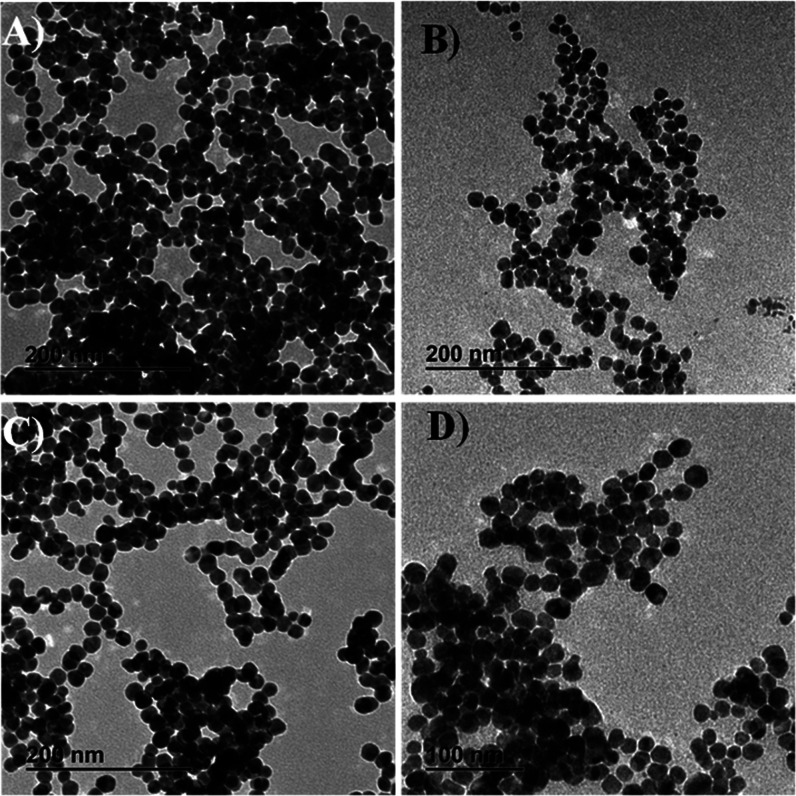
Representative TEM images for Ag NPs obtained after 4 and 6 h of reaction using the T_aquo_ extract (A,B) and after 4 and 6 h of reaction using the T_hydro_ extract and (C,D) without an incubation period.

As was previously mentioned, the synthesis of Ag NPs was carried out by applying an incubation step of 12 h to the just synthesized Ag NPs, based on previous reported protocols that point out the benefit of an incubation time in the Ag NPs quality.^19,53,66^ Figure 4 includes representative TEM images for Ag NPs obtained after 4 and 6 h of reaction using both the T_aquo_ and the T_hydro_ extracts with the mentioned incubation period. As observed, well-dispersed and very spherical Ag NPs were obtained in all cases. For the syntheses performed during 4 h of reaction, Figure 4A for T_aquo_ and Figure 4C for T_hydro_, the obtained Ag NPs are very spherical, as are those obtained after 6 h of reaction, Figure 4B for T_aquo_ and Figure 4B for T_hydro_ extracts.

**Figure 4:**
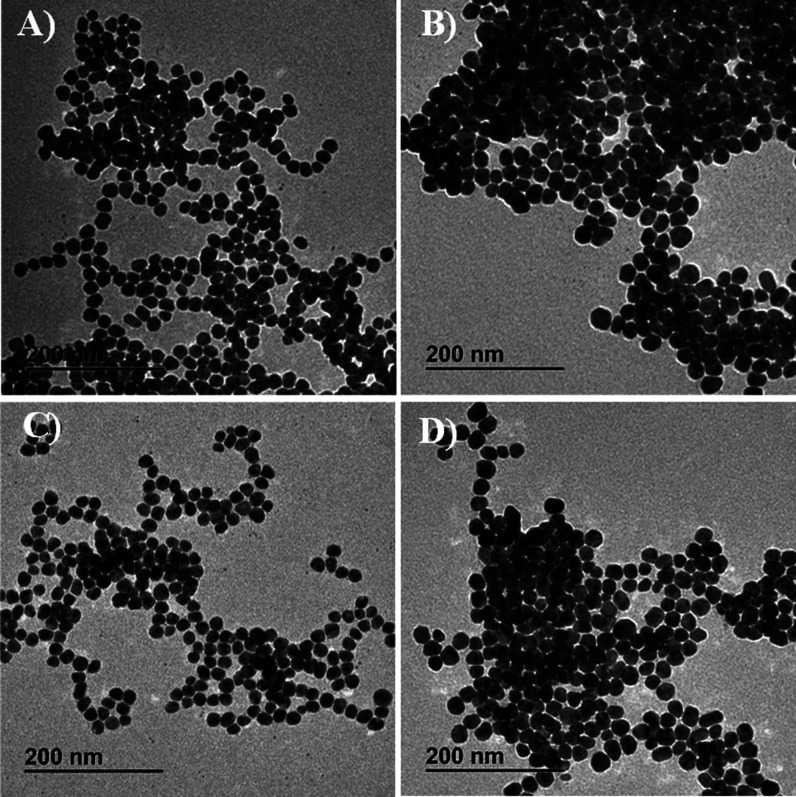
Representative TEM images for Ag NPs obtained after overnight (12 h) incubation during 4 and 6 h of reaction using the T_aquo_ extract (A,B) and during 4 and 6 h of reaction using the T_hydro_ extract (C,D).

The average particle size and the particle size distribution histograms for Ag NPs obtained after 4 and 6 h of reaction using the T_aquo_ extract with an incubation period are included in Figure S6A,B. These values were measured as 21.3 ± 2.1 and 23.9 ± 2.9 nm, respectively. Figure S6C,D show the average particle size and the particle size distribution histograms for Ag NPs obtained during 4 and 6 h of reaction using the T_hydro_ extract and the incubation period, resulting in 20.5 ± 2.0 and 22.2 ± 2.8 nm, respectively. After the incubation step, the increase of reaction time only produced a small increase in the average Ag N size and in the particle polydispersity. As in the previous case, the standard deviation suggested that the more convenient Ag NPs are obtained at 4 h of reaction with an incubation period of 12 h.

#### 3.1.4 Effect of the Reaction Temperature

Finally, for the green synthesis of Ag NPs, the effect of the reaction temperature has been widely investigated.^19,60,66^ Here, we performed the synthesis of Ag NPs at two different temperatures, 60 and 80 °C. It is important to mention that in both cases (T_aquo_ and T_hydro_), the synthesis was developed at a pH value of 10.0, during 4 h, allowing a 12 h incubation period. Figure 5A,B include representative TEM images of Ag NPs synthesized at 60 and 80 °C using the T_aquo_ extract.

**Figure 5:**
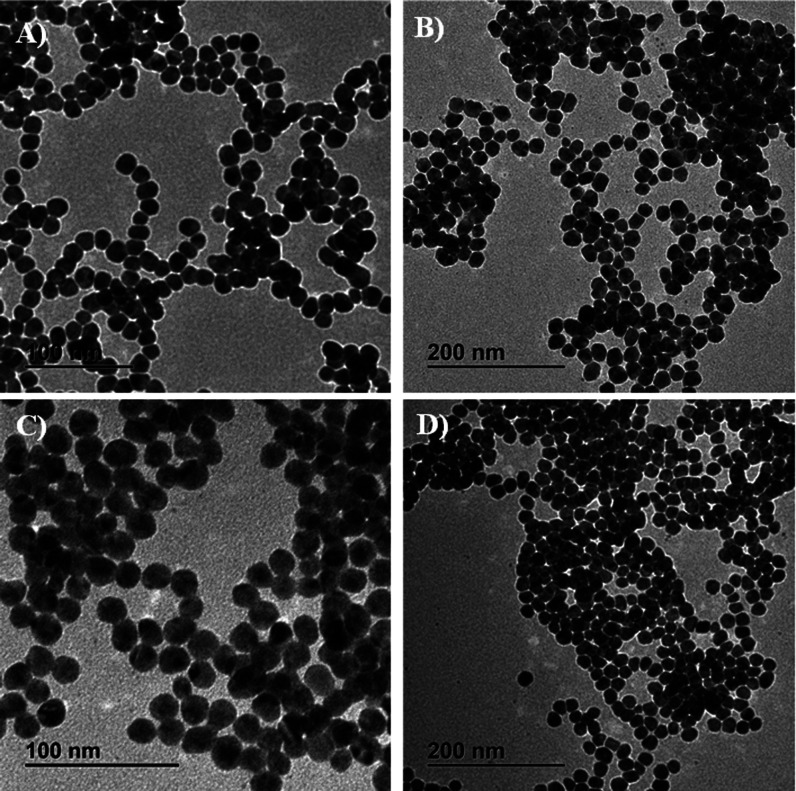
Representative TEM images for Ag NPs synthesized using the T_aquo_ extract at (A) 60 and (B) 80 °C and using the T_hydro_ extract at (C) 60 and (D) 80 °C.

As observed, well-distributed and highly monodisperse spherical Ag NPs were obtained. The average particle size and the particle size distribution histograms are included in Figure S7A,B, obtaining 21.3 ± 2.1 and 17.9 ± 2.5 nm, respectively. The average particle size decreases as the temperature of the reaction increases. Figure 5C,D include representative TEM images for Ag NPs obtained at 60 and 80 °C using the T_hydro_ extract. Again, well-distributed and spherical Ag NPs were obtained in both cases. Figure S7C,D includes the average particle size and the particle size distribution histograms, which were measured as 20.5 ± 2.0 and 18.1 ± 2.3 nm. The average particle size decreases as a function of the temperature of the reaction, obtaining a more monodisperse distribution of Ag NPs at 60 °C.

After this exhaustive screening of experimental conditions for both extracts, the optimized conditions to obtain highly monodisperse, well-dispersed, and very spherical Ag NPs, avoiding the presence of undefined larger morphologies, were a pH value of ca. 10.0, conducting the reaction for 4 h at 60 °C, and allowing an incubation period of 12 h. Table 1 summarizes the average particle size and standard deviation for Ag NPs obtained under the investigated conditions using the T_aquo_ and the T_hydro_ extracts.

**Table 1:** Particle Average Size and Standard Deviation for Ag NPs Obtained at Different Conditions Using the T_aquo_ and the T_hydro_ Extracts

	pH		without incubation		with incubation		temperature	
	9.5	10.3	4 h	6 h	4 h	6 h	60°C	80°C
T_aquo_	18.9 ± 3.7	18.2 ± 3	20.2 ± 2.9	20.8 ± 3.3	21.3 ± 2.1	23.9 ± 2.9	21.3 ± 2.1	17.9 ± 2.2
	9.0	10.0	4 h	6 h	4 h	6 h	60°C	80°C
T_hydro_	20.8 ± 3.8	20.3 ± 3.7	19.8 ± 3.1	20.2 ± 5.5	20.5 ± 2.0	22.2 ± 2.8	20.5 ± 2.0	18.1 ± 2.3

To the best of our knowledge, our protocol produces one of the most spherical and monodisperse Ag NPs mediated by a plant extract, as described in the literature. Table S1 provides all the reported protocols concerning the synthesis of Ag NPs by using different plant extracts, including the parameters investigated, the range of sizes, and the standard deviations obtained.

The optimized Ag NPs were also submitted to SEM–EDX analysis. Figure S8 shows an SEM image and an EDX spectrum for the Ag NPs obtained using the T_aquo_ extract. As observed, the SEM image shows well-dispersed, spherical, and monodispersed Ag NPs, and the EDX spectrum, acquired inside the square white area, confirms the presence of Ag. The EDX spectrum also indicates the presence of C and O, which probably come from the capping biomolecules. The analysis of the elemental composition is also included in Figure S8, obtaining 78.4% Ag, 17.4% C, and 4.2% of O. Figure S9 includes the SEM and EDX analyses for Ag NPs obtained with the T_hydro_ extract. Again, the SEM image shows spherical and monodispersed Ag NPs, and the EDX analysis confirms the presence of Ag, together with C and O. The elemental composition analysis inside the area marked with a white square resulted in 83.5% of Ag, 13.5% of C, and 3.0% O. As expected, the composition of both Ag NPs obtained under the play of each extract is very similar, and almost no variations in the elemental composition of the biomolecules present on the surface are also observed.

To confirm the presence and nature of the capping agents, we conducted FTIR analysis on isolated Ag NPs produced by using both plant extracts. The spectra for T_hydro_ and T_aquo_ AgNPs (Figures S10–S12) exhibited a markedly similar spectroscopic profile, featuring nearly the same number of bands, albeit with varying intensities. Both spectra displayed characteristic stretching bands for C=O (at 1722 cm^–1^), assigned to carbonylic moieties probably coming from carboxylic acids or esters, asymmetric/symmetric stretching for Csp^3^–H (at 2953, 2914, and 2849 cm^–1^) from aliphatic groups, and asymmetric/symmetric bending frequencies of aliphatic C–H bands at 1373 and 1267 cm^–1^, all of them contained in polyphenolic compounds as model compounds of multidentate capabilities and also reducing or antioxidant activity. Additionally, the FTIR spectrum revealed several overlapping N–H and O–H vibration modes centered at 3369 cm^–1^, likely arising from oxidized alcoholic and phenolic compounds. Furthermore, we identified C=C stretching bands belonging to acyclic and cyclic compounds at 1881 and 1355–1373 cm^–1^, respectively, that again constitute the skeleton of most of the bioactive compounds present in plant extracts. Figures S10 and S11 present each FTIR spectrum for each extract, and Figure S12 presents an overlay of the two spectra, where specific bands at 2351, 2313, and 1499 cm^–1^ were exclusively observed in the T_hydro_ sample, while the band at 1655 cm^–1^ was solely present in T_aquo_. This suggests that some compounds may differ on the surfaces of the Ag NPs produced in the two studied extracts.

The crystalline nature of the synthesized Ag NPs was confirmed through the analysis of the single-crystal diffraction pattern. Figure S13 shows the selected area electron diffraction pattern recorded from the Ag NPs fabricated with the T_aquo_ and the T_hydro_. The bright spots located in ring-like diffraction patterns correspond to (111), (200), (220), and (311) Bragg reflection planes,^77^ which could be indexed on the basis of the fcc structure of silver. The patterns show that the crystals are mostly oriented on the (111) plane, and due to this, a sharper and more intense ring is observed in the (111) plane.

### 3.2 Stability Study as a Function of pH

As mentioned, the synthesis of Ag NPs by green chemistry enables the fabrication of nanoparticles with potential applications in biomedical fields. A multitude of reported methodologies employ plant extracts, which provide numerous biomolecules and natural compounds that possess in their structure functional groups that are responsible for the reduction process (from Ag^+^ to Ag^0^). Some of these molecules can be incorporated onto the Ag NP surface and provide colloidal stability. For this reason, with the aim of providing the stability of our synthesized Ag NPs and the presence of stabilizing molecules, we include a stability investigation performed at several pH values. This stability assay is compared with Ag NPs obtained by using NaBH_4_ as a reducing agent. In this sense, colloidal stability is demonstrated by acquiring the UV–vis spectra and TEM images of Ag NPs dispersion as a function of pH. Figure S14A includes the evolution of the pH value of an Ag NP dispersion synthesized with the T_aquo_ extract after the addition of several aliquots of NaOH 0.1 M. The pH increases from 7.7 to 11.3 and, interestingly, the normalized UV–vis spectra acquired at every pH value remain constant, thus suggesting total particle stability and the absence of particle aggregation (Figure S14B). The same stability assay was performed with the Ag NPs obtained in the presence of the T_hydro_ extract. Figure S15A shows the evolution of the pH value in Ag NPs dispersion obtained using the T_hydro_ extract after the addition of several amounts of NaOH 0.1 M. In this case, the pH increases from 7.6 to 12.1. Again, the normalized UV–vis spectra do not show evidence of particle aggregation (Figure S15B). In both cases, it is evident that the molecules incorporated on the Ag NP surface provide enough chemical stability to avoid particle aggregation. This is confirmed by acquiring TEM images of the Ag NP dispersion after the addition of different amounts of NaOH. Figures S16 and S17 include TEM images at several pH values for Ag NPs obtained in the presence of the T_aquo_ and the T_hydro_ extracts, respectively. Well-dispersed and homogeneous Ag NPs with the absence of particle aggregation can be observed in both cases. The situation changes when the pH is increased in an Ag NP dispersion synthesized using NaBH_4_ as the reducing agent. Figure S18A includes the evolution of the pH value of this Ag NP dispersion after the addition of several aliquots of NaOH 0.1 M. In this case, the pH increases from 8.2 to 12.1. However, the normalized UV–vis spectra show that the Ag NPs are stable only at a pH value of 8.2, and above this pH value, the UV–vis considerably decreases, thus suggesting particle aggregation and the loss of colloidal stability (Figure S18B). This aggregation is confirmed in Figure S19, which includes TEM images of Ag NPs obtained using NaBH_4_ and after the addition of several amounts of NaOH. As observed, the presence of clusters and particle aggregations demonstrate the loss of particle stability.

### 3.3 NMR Characterization of the Metabolites from T_aquo_ and T_hydro_ Extracts Involved in Ag NP Synthesis

As already reported, *T. ramosissimum* extracts contain a multitude of organic compounds, such as flavonoids, monoterpenoids, sesquiterpenes, hydroxycinnamic derivatives, phenolic acids, fatty acids, and polysaccharides.^52,54,74,78,79^ Therefore, understanding the key molecules involved not only in the reducing process but also in the capping-assisted process is crucial for gaining insights into the generation of such spherical monodisperse Ag NPs. It is important to mention that capping molecules are the first line of interaction between the nanoparticles and the components of the medium and could also interfere with the antibacterial activity.^80^ In fact, it has been previously demonstrated that nanoparticles coated with polymers or organic compounds generate systems with low or no cytotoxicity against mammalian cells without reducing the toxic effects against bacteria assayed.^81^

To shed light on this matter, we conducted NMR investigations to perform a tentative assignment of the role of specific molecules in the reduction process and, subsequently, in the production of Ag NPs. By unraveling the molecular mechanisms underlying Ag NP synthesis, we pave the way for enhanced control and handling of these nanoparticles, opening up a multitude of possibilities for their application in various fields. Figure 6 shows a compelling comparison between the T_aquo_ and T_hydro_ extracts before (blank) and after (down) the synthesis of Ag NPs.

**Figure 6:**
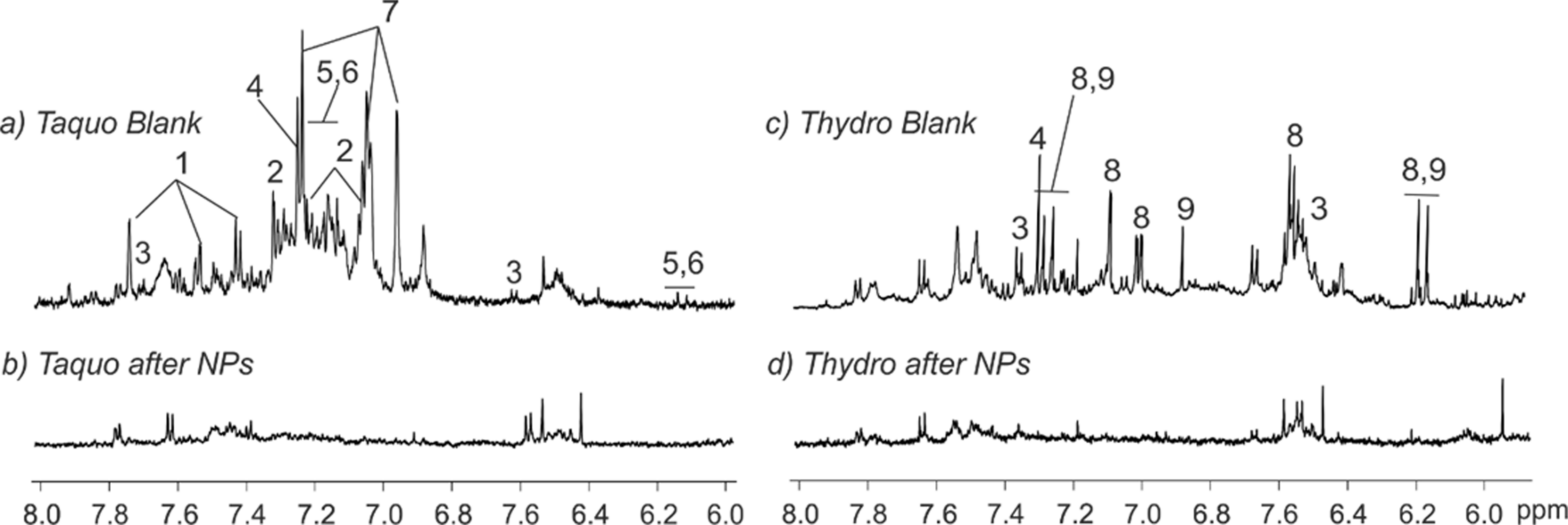
Downfield region of the ^1^H NMR spectra (600 MHz, δ_H_ 6.2 to 8.2 ppm) from the T_aquo_ extract of *T. ramosissimum* (a) before (blank) and (b) after the synthesis of Ag NPs and from the T_hydro_ extract (c) before (blank) and (d) after the synthesis of Ag NPs. Numbers identify some of the compounds for which the content decreased during the Ag NP synthesis in this spectral region: **1**: protocatechuic acid, **2**: gentisic acid, **3**: 4-hydroxybenzoic acid, **4**: gallic acid, **5**, **6**: phenylpropanoids probably of the type of chlorogenic acids, **7**: 3,4-dihydroxy-β-phenylethanol, **8**: caffeic acid, and **9**: sinapic acid.

Notably, a significant reduction in the intensity of peaks located in the downfield region of the NMR spectra, predominantly associated with phenolic compounds, was evidenced. The latter statement strongly indicates the active involvement of these compounds in the reduction process, leading to the formation of Ag NPs. Phenolic compounds are generally considered potential bioreducing and stabilizing agents that could lead to the formation of Ag NPs through the interactions established between the multipodant molecules existing in the biomass with silver metal, whether ions or elemental silver.^45^ In T_aquo_ NMR spectra, these compounds were mainly assigned to four phenolic acids: protocatechuic acid (**1**), gentisic acid (**2**), 4-hydroxybenzoic acid (**3**), and gallic acid (**4**) (Scheme 1). Furthermore, the NMR spectra show the signals from *trans*-olefinic protons at δ_H_ 7.63 and δ_H_ 6.43 ppm, which were also deduced and assigned to phenylpropanoids (**5**, **6**), probably of the type of chlorogenic acids (see below). Finally, a 2-phenylethanol derivative could also be identified out of the full spectroscopic profile, which was tentatively assigned to 3,4-dihydroxy-β-phenylethanol (**7**) due to some ^13^C NMR signals located at δ_C_ 127.0, 124.2, 124.9, and 29.2 ppm identified in the two-dimensional ^1^H–^13^C HSQC edited HSQC spectrum.

**Scheme 1:**
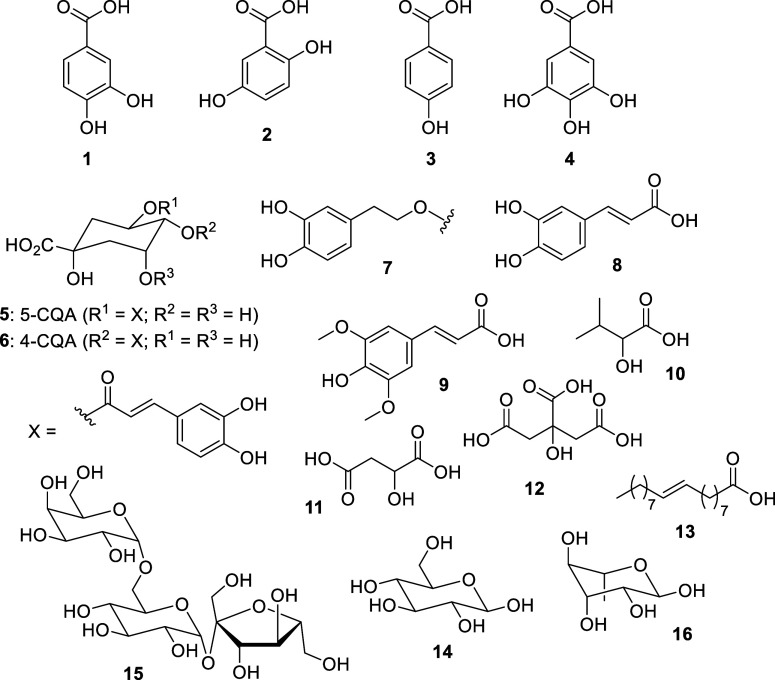
Active Molecules Involved in the Ag NPs Synthesis

Regarding the T_hydro_ extract, several key species were identified to participate in the synthesis of Ag NPs. Notably, two phenylpropanoids were identified, with a caffeic acid derivative (**8**) being the predominant species, followed by sinapic acid (**9**), and an unidentified compound (**10**). Furthermore, the T_hydro_ spectra revealed the presence of gallic acid (**4**) and 4-hydroxybenzoate (**5**) (Scheme 1).

Regarding protocatechuic acid (**1**), gentisic acid (**2**), and gallic acid (**4**), it is noteworthy that gallic acid, in particular, is widely recognized as one of the most prevalent phenolic acids found within the plant kingdom. Indeed, its presence has been documented in various *Teucrium* species. The compound 4-hydroxybenzoic acid (**3**) has been described as being present in *Teucrium arduini* flower and leaf extracts.^82^ Besides simple phenolic acids, several representatives of hydroxycinnamic acids or phenylpropanoids and their derivatives, mostly caffeic (**8**), ferulic, and *p*-coumaric acids, have also been identified in a notably high number of *Teucrium* plant species, whereas chlorogenic (**5**, **6**) and sinapic (**9**) acids have been screened out of ten and four *Teucrium* species, respectively.^82^ On the contrary, compound 3,4-dihydroxy-β-phenylethanol (**7**) has been exclusively identified in *Teucrium hyrcanicum*.^78^ The most common phenylethanoid is verbascoside, a caffeoyl phenylethanoid glycoside in which the phenylpropanoid caffeic acid and the phenylethanoid hydroxytyrosol are coupled to the rhamnose carbohydrate of rutinose (Scheme 1). This compound has been found in 12 investigated plants from the *Teucrium* genus, and it is usually present in higher concentrations compared with flavonoids and phenolic acids.^82^ However, in our case, we could not find the corresponding phenylpropanoid part of the molecule in the spectra at the same peak integral, so we believe that (**7**) may come from a phenylethanoid glycoside with the typical phenethyl alcohol moiety attached to a β-glucopyranose/β-allopyranose (such as leonoside E, which was found in polar fractions of *Teucrium sandrasicum* O. Schwarz roots.^83^

Concerning the low-frequency region of the NMR spectra, 2-hydroxyisovaleric acid (**10**) (for T_aquo_) and malic acid (**11**) (for both T_aquo_ and T_hydro_) were also shown to be involved in Ag NP formation (Scheme 1). Typically, these acids serve as both reducing and coordinating agents. However, it is worth noting that they are often employed independently in synthetic pathways, which may not necessarily align with the principles of green chemistry.^84^ Interestingly, peaks from citric acid (**12**) also disappeared in the spectra of T_aquo_ suggesting a reducing role in the synthesis or a stabilizing interaction with the forming Ag NPs (see HS-SPME-GC-qMS section below). Indeed, some signals belonging to fatty acids (**13**), especially unsaturated fatty acids (UFA), were also found to decrease with Ag NP synthesis with T_aquo_. Fatty acids, such as myristic, palmitic, stearic, and oleic acids from vegetable oils, have been previously used in the synthesis of Ag NPs as stabilizers due to their amphiphilic nature: their polar carboxylic groups are able to coordinate with Ag NPs, while their nonpolar long carbon chains can prevent NP aggregation through steric repulsion.^85,86^ Finally, some sugars that, according to literature, were identified as glucose (**14**), raffinose (**15**), and rhamnose (**16**),^79^ also disappeared from the spectra in T_hydro_ extract.^79^ Tables 2 and 3 show the chemical assignments for the above-mentioned compounds found in *T. ramosissimum* aqueous (T_aquo_) and hydroethanolic (T_hydro_) extracts. All the compounds were assigned based on 2D experiments (^1^H–^1^H TOCSY, ^1^H–^1^H COSY, ^1^H–^13^C HMBC, and ^1^H–^13^C HSQC) and literature. Certain assigned metabolites may exist in the form of their glycoside derivatives; however, we were unable to determine the specific sugar components linked to them. Although ^1^H chemical shifts of the same compound can vary due to anisotropic effects as a function of the solvent used, i.e., hydroethanolic versus aqueous, ^13^C NMR is almost unaffected and therefore validated our assignments.

**Table 2:** Peak Assignments of the Metabolites from *T. ramosissimum* Aqueous Extract (T_aquo_) Identified to be Involved in the AgNP Synthesis

metabolite	assignment	^1^H (ppm)	mult, *J*_Hz_	^13^C (ppm)
protocatechuic acid	CH-2	7.84	d, *J* = 2.0 Hz	124.6
	CH-6	7.65	dd, *J* = 7.9, 2.0 Hz	133.4
	CH-5	7.52	d, *J* = 7.9 Hz	124.5
	COOH			171.0
gentisic acid	CH-6	7.42	d, *J* = 1.8 Hz	124.6
	CH-4	7.33	dd, overlap	128.8
	CH-5	7.16	d, overlap	124.0
	COOH			173.2
4-hydroxybenzoate	CH-2, CH-6	7.83	d, overlap	132.0
	CH-3, CH-5	6.72	d, *J* = 8.7 Hz	119.7
	COOH			170.2
gallic acid	CH-2, CH-6	7.35	s	109.1
	C-4			154.2
	C-7, COOH			168.9
phenylpropanoid 1	CH-8′ (=CH–COO)	6.23	d, *J* = 15.0 Hz	
	CH-7′ (–CH=)	7.33	d, *J* = 15.0 Hz	
	CH-2′	7.15	d, *J* = 1.9 Hz	
	CH-6′	7.07	dd, *J* = 8.0, 1.9 Hz	
	CH-5′	6.64	d, *J* = 8.0 Hz	
phenylpropanoid 2	CH-8′ (=CH–COO)	6.23	d, *J* = 15.1 Hz	
	CH-7′ (–CH=)	7.33	d, *J* = 15.1 Hz	
3,4-dihydroxy-β-phenylethanol		7.35	d, *J* = 8.4 Hz	127.0
		7.16	dd, *J* = 8.4, 1.7 Hz	124.2
		7.08	d, *J* = 1.7 Hz	124.9
		2.87	b.s.	29.2
citric acid	α,γ-CH	2.70	d, *J* = 15.1 Hz	41.8
	α′,γ′-CH	2.55	d, *J* = 15.1 Hz	
	β-C			72.9
	1,5-COOH			176.4
	6-COOH			178.6
malic acid	β,β′-CH_2_	2.36	dd, *J* = 15.1; 10.0 Hz	42.7
		2.67	dd, *J* = 15.1; 3.0 Hz	
	α-CH	4.30	dd, *J* = 10.0; 3.0 Hz	70.7
	–COOH			180.3
2-hydroxyisovalerate	γ-CH_3_	0.90 1.01 2.15	d, *J* = 7.3 Hz	16.2
	γ′-CH_3_	4.10	d, *J* = 7.3 Hz	17.8
	β′-CH		m	32.5
	α-CH		d, overlap	83.2
				178.1
unsaturated fatty acids	–CH=CH–	5.40	b.s.	128.1
	=CH–CH_2_–CH=	2.86	b.s.	26.5
	–CH_2_–CH_2_–COOR	1.55	b.s.	26.7

**Table 3:** Peak Assignments of the Metabolites from the *T. ramosissimum* Hydroethanolic Extract (T_hydro_) Identified to be Involved in AgNP Synthesis

metabolite	assignment	^1^H (ppm)	mult, *J*_Hz_	^13^C (ppm)
caffeic acid derivative	COOH			177.0
	CH-8′ (=CH–COO)	6.23	d, *J* = 15.0 Hz	117.4
	CH-7′ (–CH=)	7.33	d, *J* = 15.0 Hz	142.7
	C-1′			120.3
	CH-2′	7.14	d, *J* = 2.0 Hz	110.8
	C-3′			151.9
	C-4′			159.6
	CH-5′	6.61	d, *J* = 8.1 Hz	118.6
	CH-6′	7.06	dd, *J* = 8.1, 2.0 Hz	124.2
sinapic acid	CH-8′ (=CH–COO)	6.25	d, *J* = 15.0 Hz	117.4
	CH-7′ (–CH=)	7.32	d, *J* = 15.0 Hz	142.7
	CH-2, CH-6	6.93	s	107.4
	C-4			150.5
	OCH_3_	3.87		
gallic acid	CH-2, CH-6	7.35	s	108.7
	C-4			154.0
	C-7, COOH			168.1
4-hydroxybenzoate	CH-2, CH-6	7.41	d, *J* = 8.8 Hz	130.2
	CH-3, CH-5	6.62	d, *J* = 8.8 Hz	118.6
	COOH			168.0
glucose	CH-1	5.28	d, *J* = 4.2 Hz	97.5
	CH-1	4.68	d, *J* = 7.5 Hz	98.2
raffinose	CH-1 (Glc)	5.42	d, *J* = 4.2 Hz	92.2
		4.17	d, *J* = 8.7 Hz	76.9
rhamnose		5.12	d, *J* = 2.2 Hz	97.4
malic acid	β,β′-CH_2_	2.36	dd, *J* = 15.0; 10.1 Hz	42.4
		2.67	dd, *J* = 15.0; 3.0 Hz	
	α-CH	4.30	dd, *J* = 10.1; 3.0 Hz	70.3
	–COOH			180.1

### 3.4 Head Space-Solid Phase Microextraction with Gas Chromatography/Quadrupole-Mass Spectrometry (HS-SPME-GC-qMS)

The fingerprints of volatile metabolites of the synthesized silver nanoparticles with both T_hydro_ or T_aquo_ under optimal analytical conditions are illustrated in Figures S20 and S21. GC–qMS chromatographic profiles revealed the presence of two main chromatographic peaks located at retention times of 15.2 and 17.1 min and of smaller intensity, another two at retention times of 14.4 and 14.5 min. The first two corresponded to the oxidation products of chlorogenic acids (CQA). Scheme 2 shows their structures together with some of the fragments that were identified in the MS spectra. The proposed compounds align with the observation that the signals of chlorogenic acids (5-CQA in Scheme 2) essentially vanished in the NMR spectra of the extracts. This implies that their oxidized derivatives likely serve as stabilizing agents, capping the surfaces of the Ag NPs. The two peaks observed at 14.4 and 14.5 min corresponded to oxidized products derived from citric acid, a well-known reducing agent widely employed in Ag NP synthesis. Polyphenolic acids or their quinonic derivatives that may have been formed were not identified on the surface of the Ag NPs, probably due to the lack of a strong interaction with the NPs that inhibits their presence after cleanup procedures. In fact, gallic acid (**4**) has already been described as a reducing and functionalizing agent for Ag NPs.^87^

**Scheme 2:**
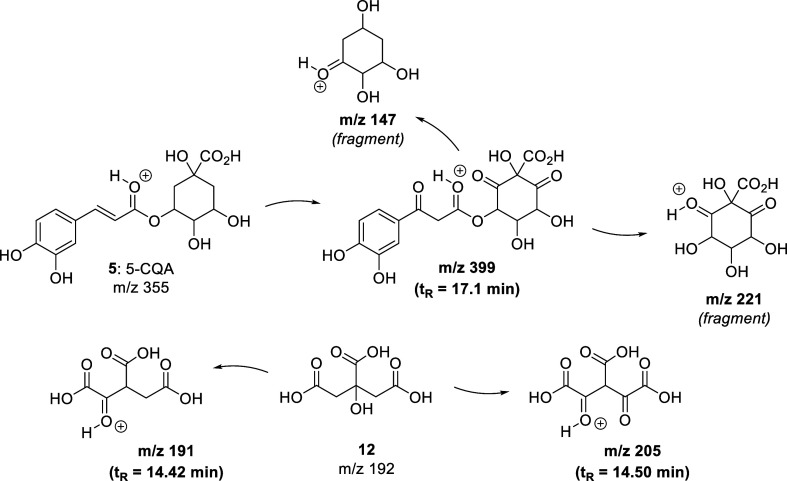
Probable Oxidation Products (Assumed to be Protonated) Originated in the Reduction of Cationic Silver in the Presence of the *T. ramosissimum* Hydroethanolic Extract

Based on the fragments observed in the several chromatographic peaks, we envisaged an oxidation mechanism where methylenic groups and conjugated double bonds undergo oxidation to form ketones and undergo hydration, respectively. These processes potentially impact the reduction of metal ions, and the resulting products effectively act as capping agents. Interestingly, similar ionized products have previously been identified in the green synthesis of silver nanoparticles using blueberry extracts.^45^

## 4 Conclusions

Here, we have performed the green synthesis of Ag NPs by using the aqueous (T_aquo_) and hydroethanolic (T_hydro_) extracts of the *T. ramosissimum*, a Lamiaceae family from the south of Tunisia. To obtain the best synthesis conditions, several parameters were investigated, such as the pH value, reaction time, reaction temperature, and the possibility of performing an overnight incubation after particle synthesis. In both cases, the well-distributed, highly spherical, and monodisperse Ag NPs were obtained by performing the synthesis at a pH value of ca. 10 at 60 °C for 4 h. Surprisingly, we observed an improvement by performing an overnight incubation after particle generation. UV–vis spectroscopy analysis and TEM investigations were used to confirm the presence of Ag NPs and analyze the size and shape of the synthesized Ag NPs. No significant differences were obtained using both extracts, only slight differences in terms of average particle size and standard deviation values. Under our optimized conditions, we probably obtain the most highly monodisperse and spherical Ag NPs reported in the literature. This offers the possibility of being used in the fabrication of 2D ordered arrays for antibacterial or sensor applications. Indeed, NMR investigations were used to determine the chemical structure of the molecules involved in the reduction process and, consequently, responsible for the generation of Ag NPs. In both cases, the NMR signals of compounds such as phenolic acids (such as protocatechuic, gentisic, 4-hydroxybenzoic, and gallic acid), phenylpropanoids (such as caffeic and sinapic acids), as well as citric and malic acid, were reduced after the synthesis. HS-SPME-GC–qMS were used to determine the molecules incorporated on the Ag NPs surface, thus resulting in 5-CQA and citric acid.

## Supplementary Material



## Notes

TEM images, TEM histograms, SEM–EDX analysis, FTIR, SAED patterns, stability assays, HS-SPME-GC–MS chromatographic profiles, and ESI-MS spectra of Ag NPs (PDF)
