# Supplementary material for: Green Synthesis of Highly Monodisperse and Spherical Ag Nanoparticles by a Combination of Teucrium ramosissimum Desf. (Lamiaceae) Extracts with Emphasis on the Stabilizing and Capping Biomolecules
Source: ACS Sustain Chem Eng. 2024 Feb 27;12(10):4132–45. doi: 10.1021/acssuschemeng.3c07504 (PMC12884677; doi:10.1021/acssuschemeng.3c07504)
Supplement: Supplementary file 1 [file sc3c07504_si_001.pdf]

Supporting Information

Green Synthesis of Highly Monodisperse and Spherical Ag Nanoparticles by a Combination of *Teucrium Ramosissimum* Desf. (Lamiaceae) Extracts with Emphasis on the Stabilizing and Capping Biomolecules

Rim Bouhajeb,[1]‡ Ana C. Abreu,[2]‡ Silvia Fernández,[2] Mohamed Bayrem-Ghedira[1], Leila Chekir-Ghedira,[1] Ignacio Fernández,[2]* Rafael Contreras-Caceres[2]*

[1] Unit of Bioactive and Natural Substances and Biotechnology UR17ES49, Faculty of Dental Medicine and Faculty of Pharmacy, University of Monastir, Monastir 5000, Tunisia.

[2] Department of Chemistry and Physics, Research Centre CIAIMBITAL, University of Almería, Almería 04120, Spain.

Table S1. Synthesis of Ag NPs using different plant and fruit extracts. **S3**

Figure S1. TEM images for Ag NPs obtained using the T_{aquo} extract at several pH values. **S4**

Figure S2. Average particle size and particle size distribution histograms for Ag NPs obtained using T_{aquo} extract at several pH values **S5**

Figure S3. TEM images for Ag NPs synthesized using 2.5 mL of T_{aquo} extract at several pH values **S6**

Figure S4. TEM images for Ag NPs synthesized using 2.5 mL of T_{hydro} extract at several pH values. **S7**

Figure S5. Average particle size and particle size distribution for Ag NPs obtained after 4 and 6 h of reaction using T_{aquo} and T_{hydro} extracts without overnight incubation. **S8**

Figure S6. Average particle size and particle size distribution for Ag NPs obtained after 4 and 6 h of reaction using the T_{aquo} extract with overnight incubation. **S9**

Figure S7. Average particle size and particle size distribution for Ag NPs obtained using the T_{aquo} and the T_{hydro} extracts at 60 and 80ºC. **S10**

Figures S8 and S9. SEM and EDX analysis of Ag NPs synthesized with the T_{aquo} and the T_{hydro} extracts. **S11**

Figures S10 and S11. FTIR for Ag NPs synthesized in the presence of the T_{aquo} and the T_{hydro} extracts. **S12**

Figure S12. FTIR comparison for Ag NPs synthesized in the presence of the T_{hydro} and the T_{aquo} extracts. **S13**

Figure S13. Selected area electron diffraction (SAED) patterns of biosynthesized silver nanoparticles with T_{aquo} and T_{hydo}. **S14**

Figure S14 and S15. Evolution of the pH and UV-vis spectra in a colloidal dispersion containing Ag NPs synthesized with the T_{aquo} and the T_{hydro} extracts after the addition of different aliquots of NaOH 0.1 M. **S15**

Figure S16. TEM images for Ag NPs obtained with the T_{aquo} extract after the addition different amounts of NaOH. **S16**

Figure S17. TEM images for Ag NPs obtained with the T_{hydro} extract after the addition different amounts of NaOH. **S17**

Figure S18. Evolution of the pH and UV-vis spectra of a colloidal dispersion containing Ag NPs synthesized with $NaBH_4$ after the addition of different amounts of NaOH 0.1 M. **S18**

Figure S19. TEM images for Ag NPs obtained using $NaBH_4$ after the addition different amounts of NaOH. **S19**

Figure S20. HS-SPME-GC-MS chromatographic profile of T_{hydro} and Ta_{quo} Ag NPs together with a blank with no Ag NPs using DVB/CAR/PDMS fibers. **S20**

Figure S21. ESI–MS (ESI+) spectrum of peaks at different retention times. **S21**

Table S1. Synthesis of Ag NPs using plant and fruit extracts. The table includes the extract used for the synthesis of Ag NPs, the parameters investigated, the nanoparticle size and the reference.

Extract	Parameters	Microscopic análisis/Particle size	Ref
Ocimum Sanctum (Tulsi) Quercetin	Incubation time, temperature, pH, concentration	Figure 9 (14.6-11.35)	19
Blueberry (*Vaccinium corybosum* L.)	pH, concentration	Figure 1 (9.9±2.4/20.5±5)	45
Blackberry, blueberry, Pomegranate, and Turmeric	Room temperature	Figures 3, 4 and 5 (200/25-150/5-50)	47
Saudi *Origanum vulgare* L.	Concetration of extract, reaction time	Figure 4 (11.27±0.32)	50
Cuphea procumbens	Reaction time	Figure 3 (12,35±2,56)	51
Pineapple *(Ananas comosus)*	Incubation time	Figure 4 (5-30 nm)	53
Capsicum annuum L.	Reaction time	Figure 2 (10±2/25 ±3/40±5/45±3)	55
Tea/epicatechin	Concentration of extract	Figure 3 (11.5±4.71/25.8±15.)	56
Garlic, green tea, turmeric	2 h, 60ºC	Figure 6 (6.13-8.46)	57
Coffe and tea	Room temperature	Figure 1	58
Hibiscus rosa	pH	Figure 7 and 8 13 nm (anisotropy)	59
Beet juice	Reaction time, temperature, concetration of extract	Figure 1-3 (20-40/10-100/10-150)	60
Black pepper (*Piper nigrum*)	Concentration of $AgNO_3$	Figure 4-8 (5-50)	61
Grape pomace	Concentration of $AgNO_3$	Figure 4 (9.3-27.9)	62
Starch	pH	Figure 2 (30-40)	65
Banana peel	Temperature, incubation time	Figure 4 (23.7±3.0)	66

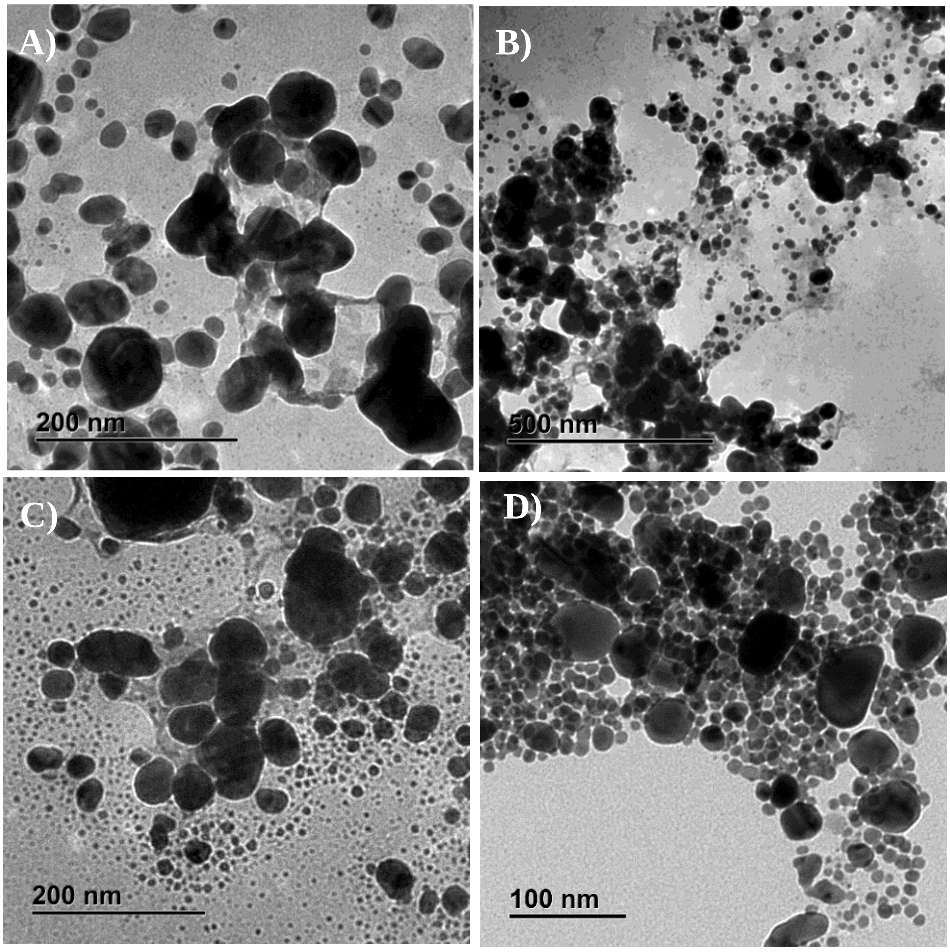

Figure S1. Representative TEM images for Ag NPs obtained using the T_{aquo} extract at pH values of A) pH 7.2, B) pH 8.8, and the T_{hydro} extract at pH values of C) pH 6.75, D) pH 8.8.

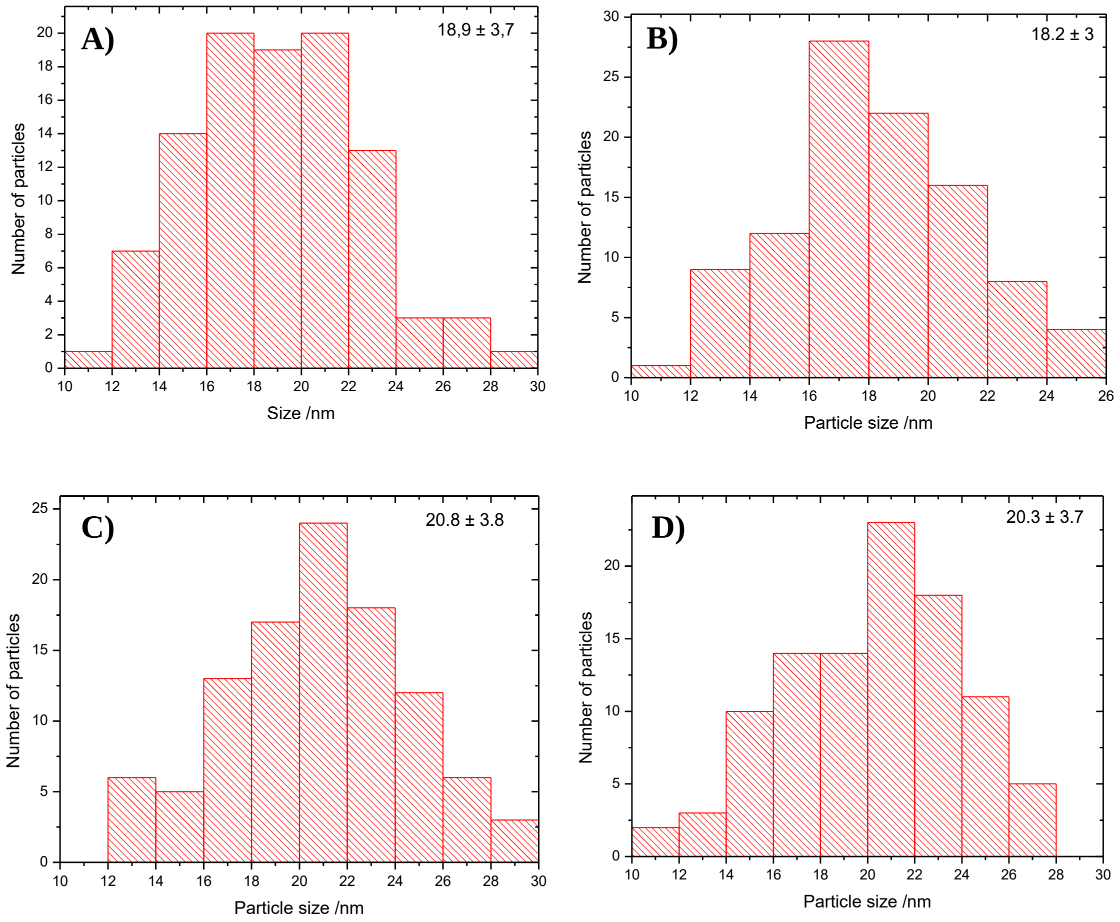

Figure S2. Average particle size and particle size distribution histograms for Ag NPs obtained using T_{aquo} extract at A) pH 9.5 and B) pH 10,3 and using the T_{hydro} extract at C) pH 9 and D) pH 10.

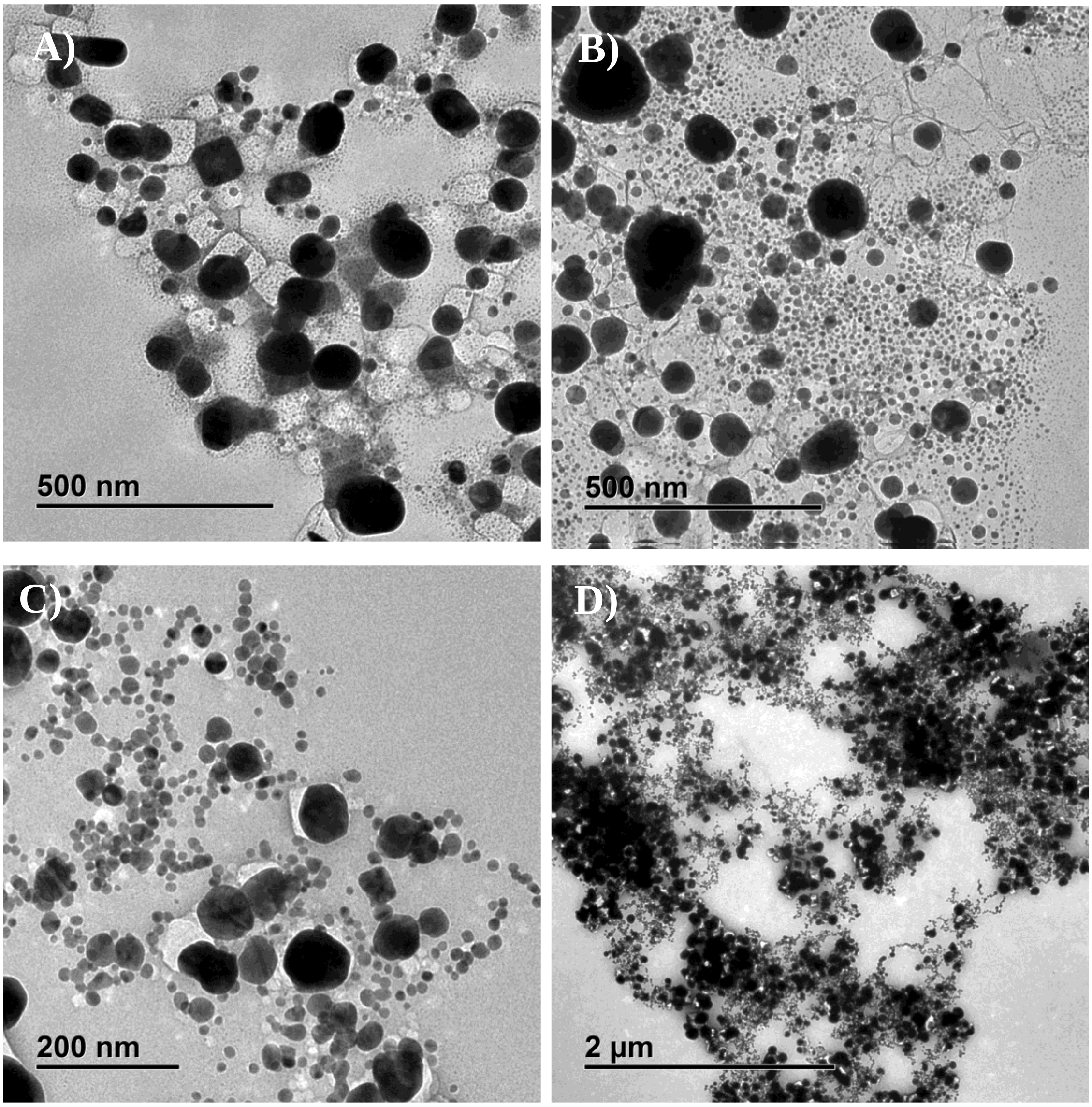

Figure S3. Representative TEM images for Ag NPs synthesized using 2.5 mL of T_{aquo} extract at pH values of A) 6.75, B) 7.3, C) 9 and D) 10.

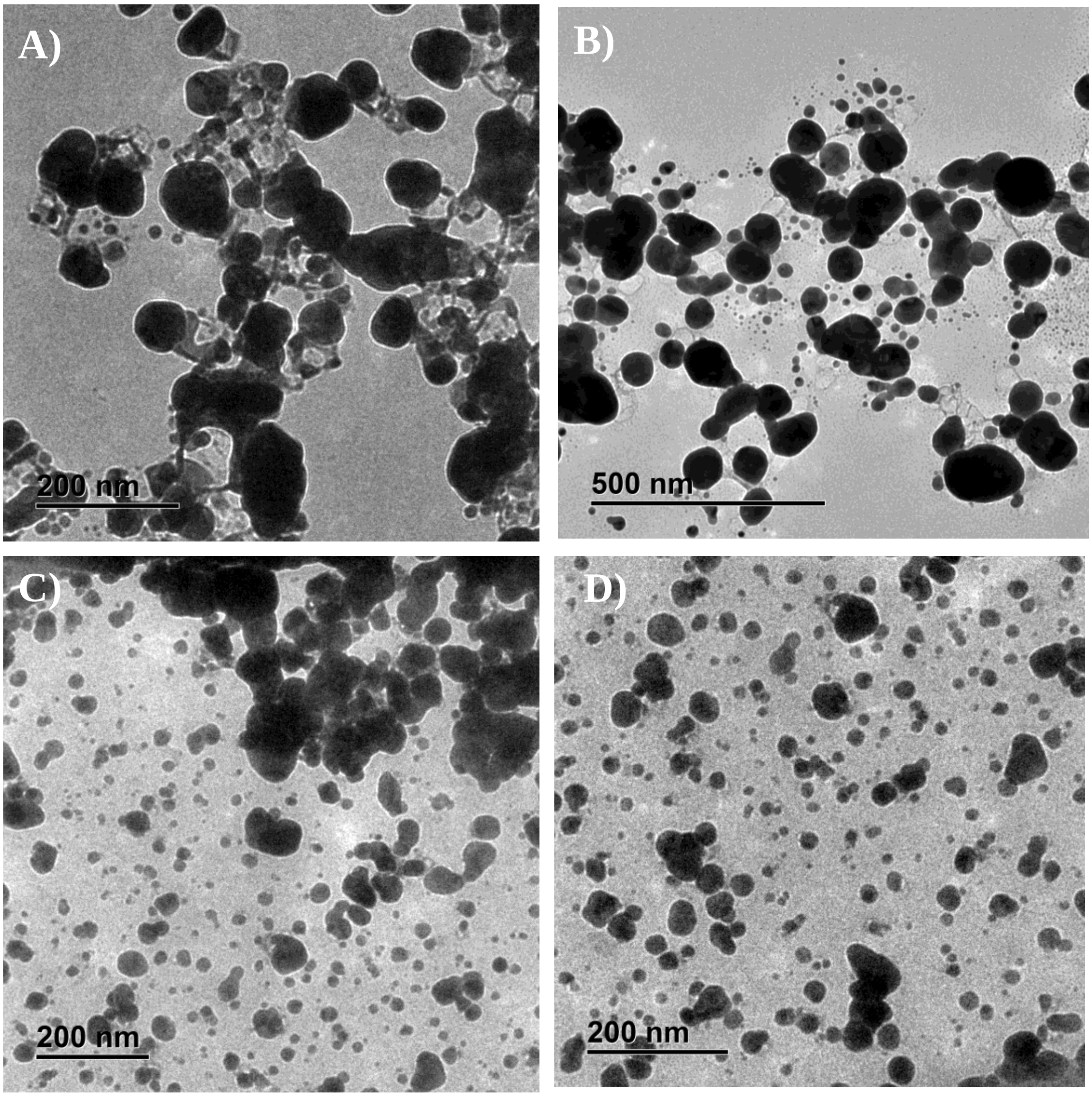

Figure S4. Representative TEM images for Ag NPs synthesized using 2.5 mL of T_{hydro} extract at pH values of A) 7, B) 8.5, C) 9 and D) 10.

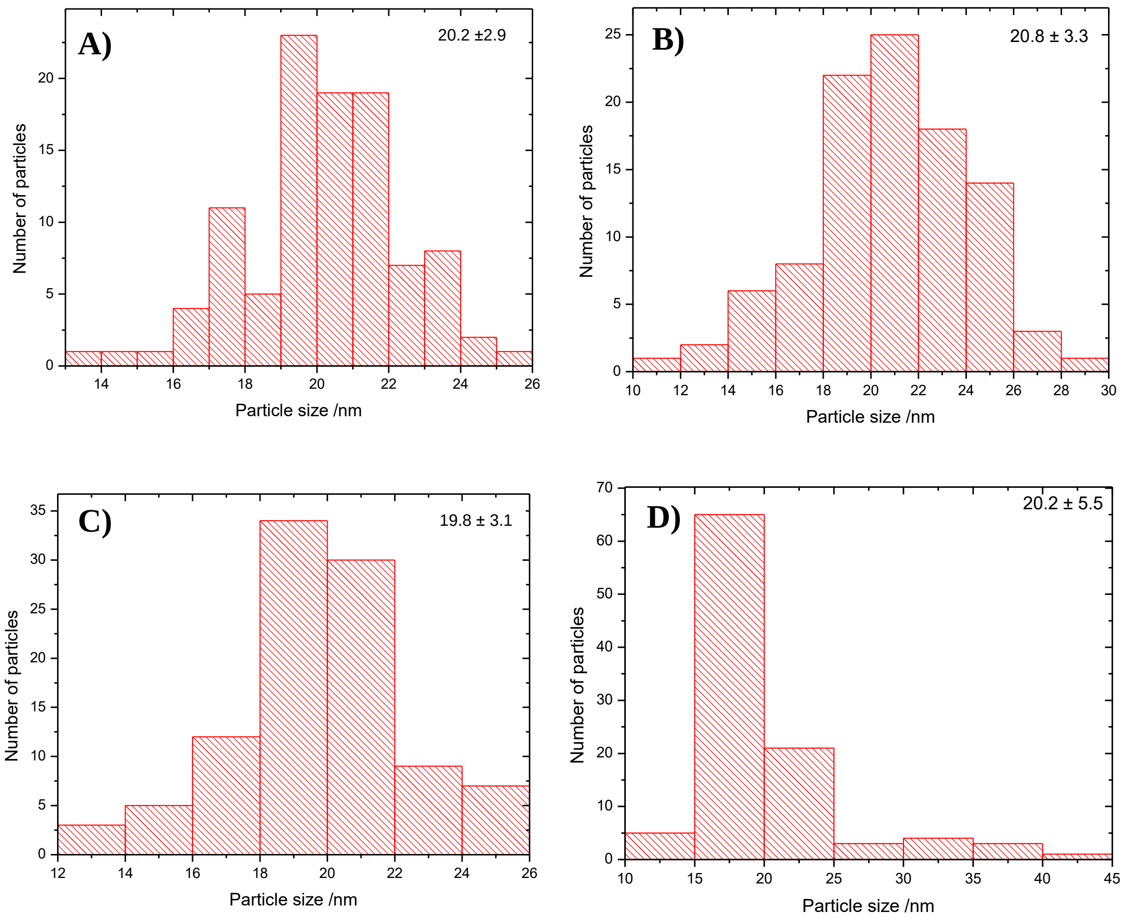

Figure S5. Average particle size and particle size distribution histograms for Ag NPs obtained after 4 h and 6 h of reaction using the T_{aquo} extract A) and B), and after 4 h and 6 h of reaction using the T_{hydro} extract and C) and D) without overnight incubation.

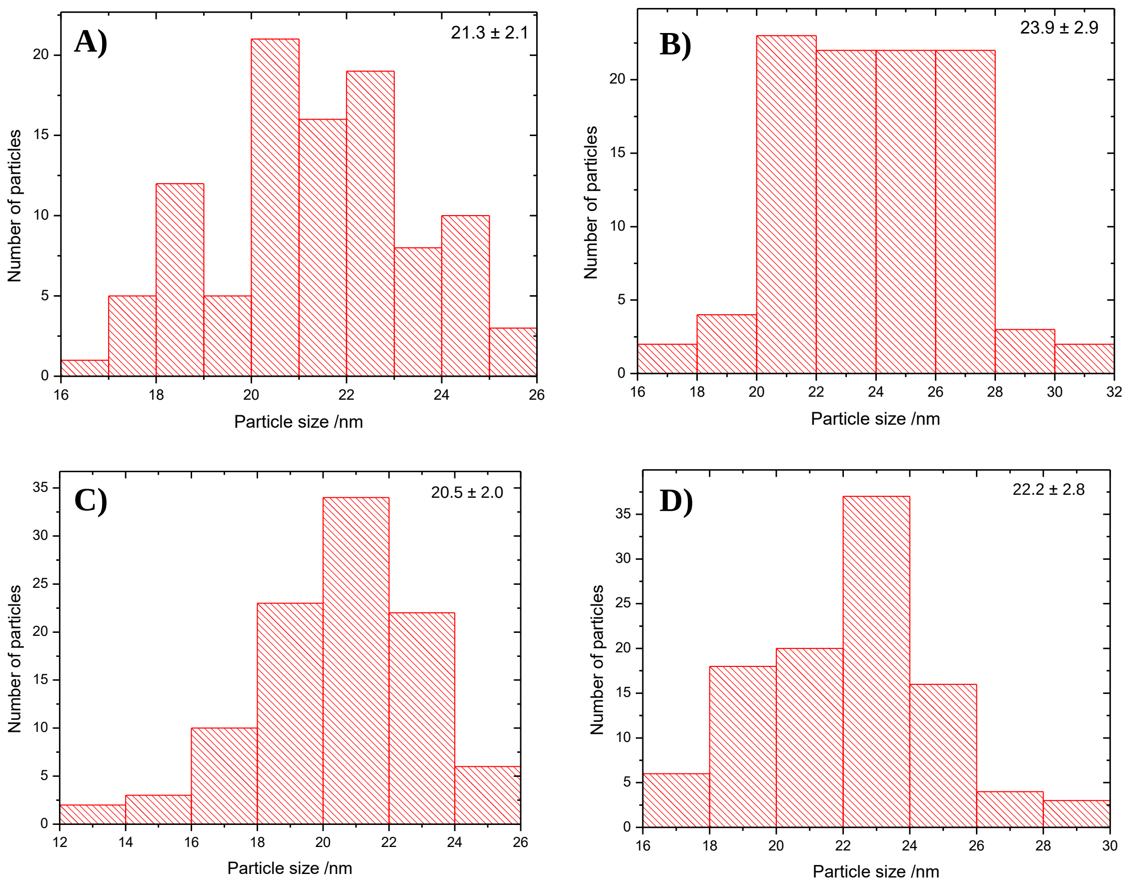

Figure S6. Average particle size and particle size distribution histograms for Ag NPs obtained after 4 h and 6 h of reaction using the T_{aquo} extract A) and B), and after 4 h and 6 h of reaction using the T_{hydro} extract and C) and D) with overnight incubation.

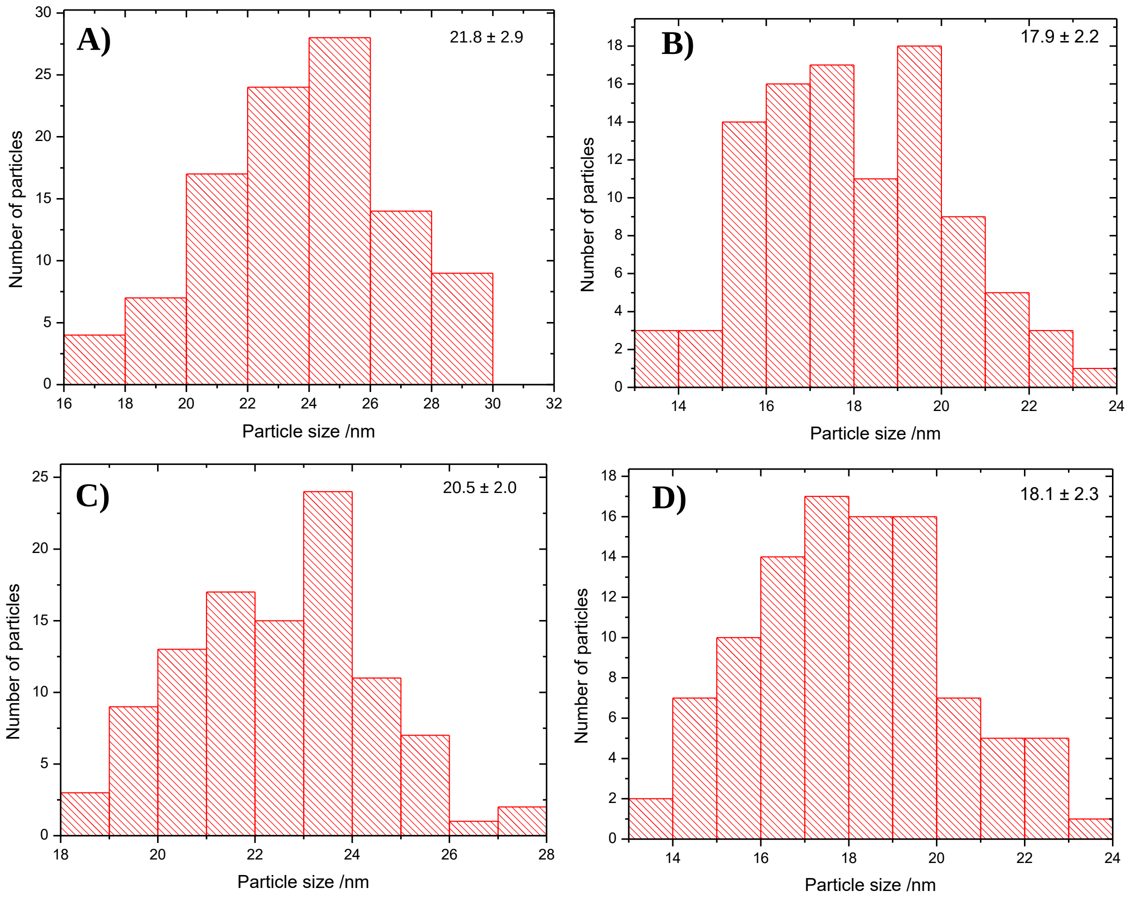

Figure S7. Average particle size and particle size distribution histograms for Ag NPs obtained using the T_{aquo} extract at A) 60 ºC and B) 80 ºC and using the T_{hydro} extract at C) 60 ºC and D) 80 ºC with particle incubation**.**

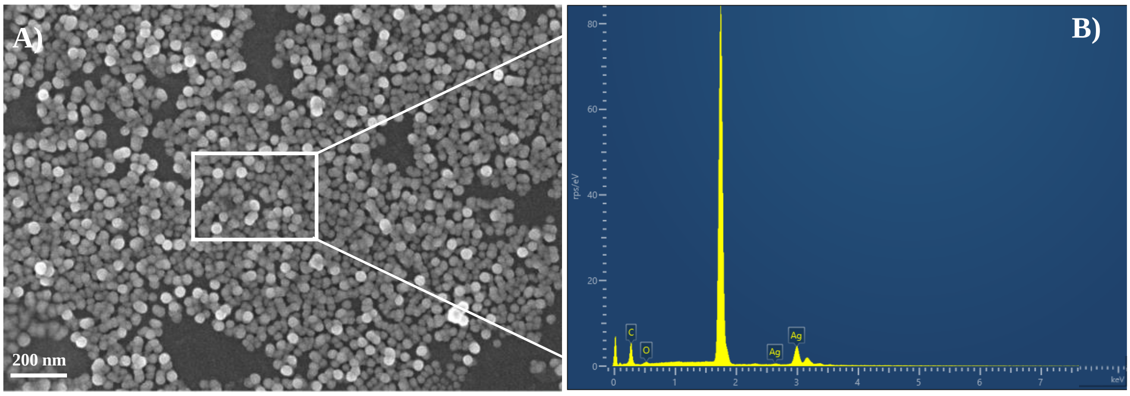

Element	Line	Elemental Composition (%)
C	K Series	17.4
O	K Series	4.2
Ag	L Series	78.4
Total:		100.00

Figure S8. A) SEM and B) EDX analysis of Ag NPs synthesized with the T_{aquo} extract. Elemental composition analysis from the area marked in the SEM image.

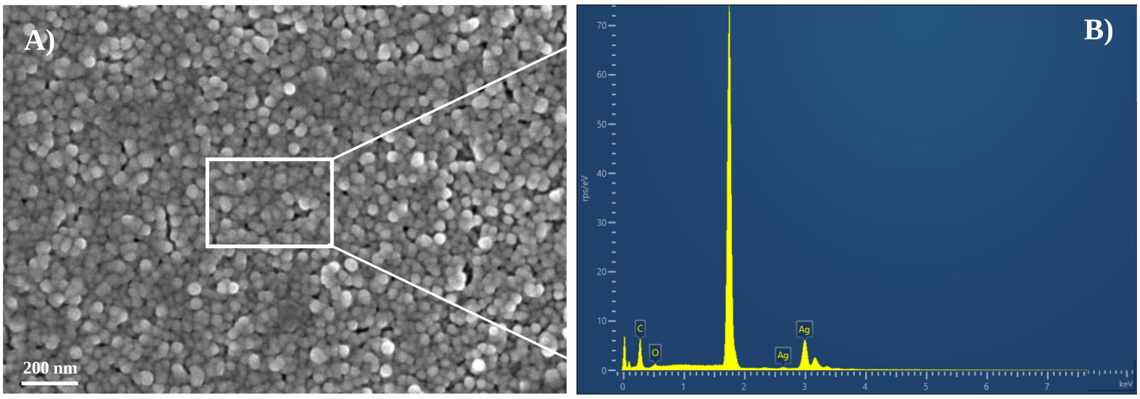

Element	Line	Elemental Composition (%)
C	K Series	13.5
O	K Series	3
Ag	L Series	83.5
Total:		100.00

Figure S9. A) SEM and B) EDX analysis of Ag NPs synthesized with the T_{aquo} extract. Elemental composition analysis from the area marked in the SEM image.

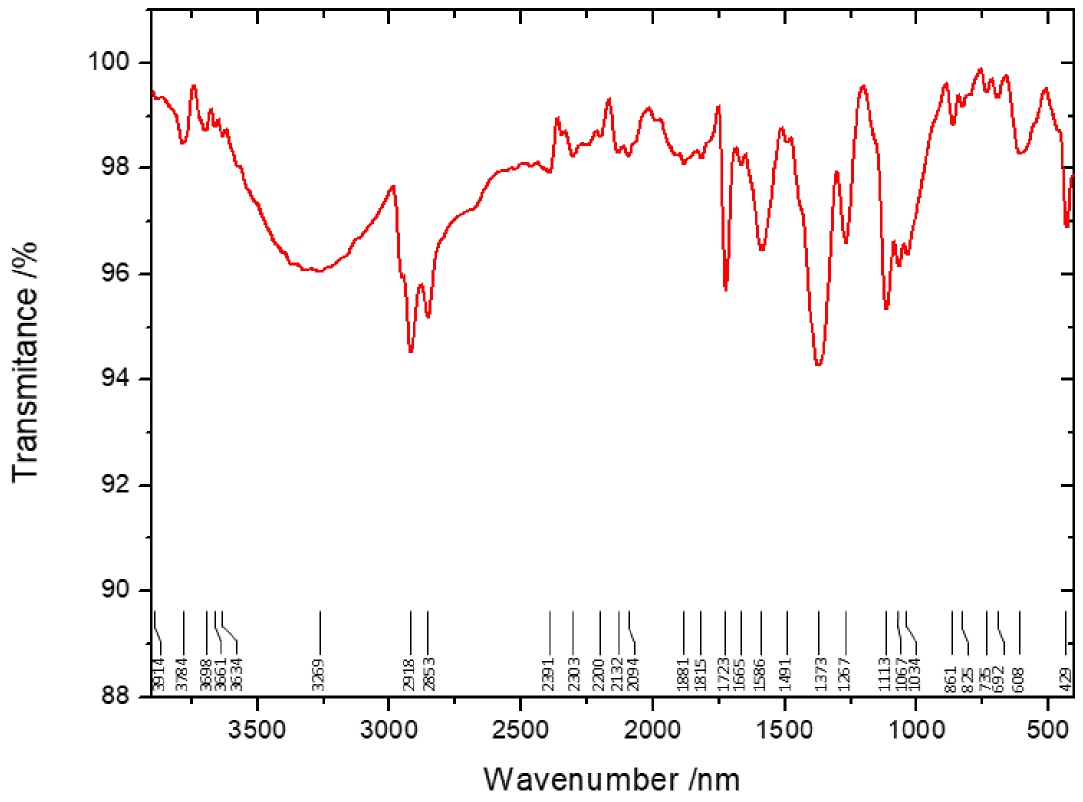

Figure S10. FTIR for Ag NPs synthesized in the presence of the T_{aquo} extract.

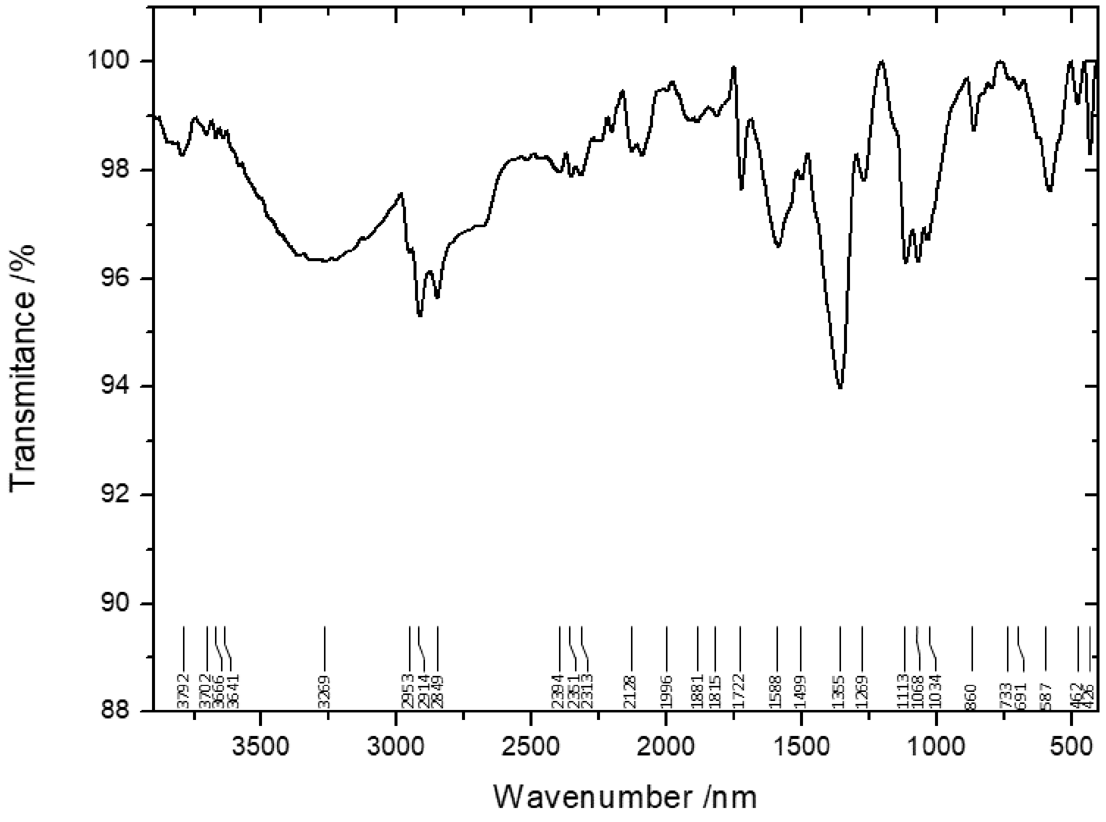

Figure S11. FTIR for Ag NPs synthesized in the presence of the T_{hydro} extract.

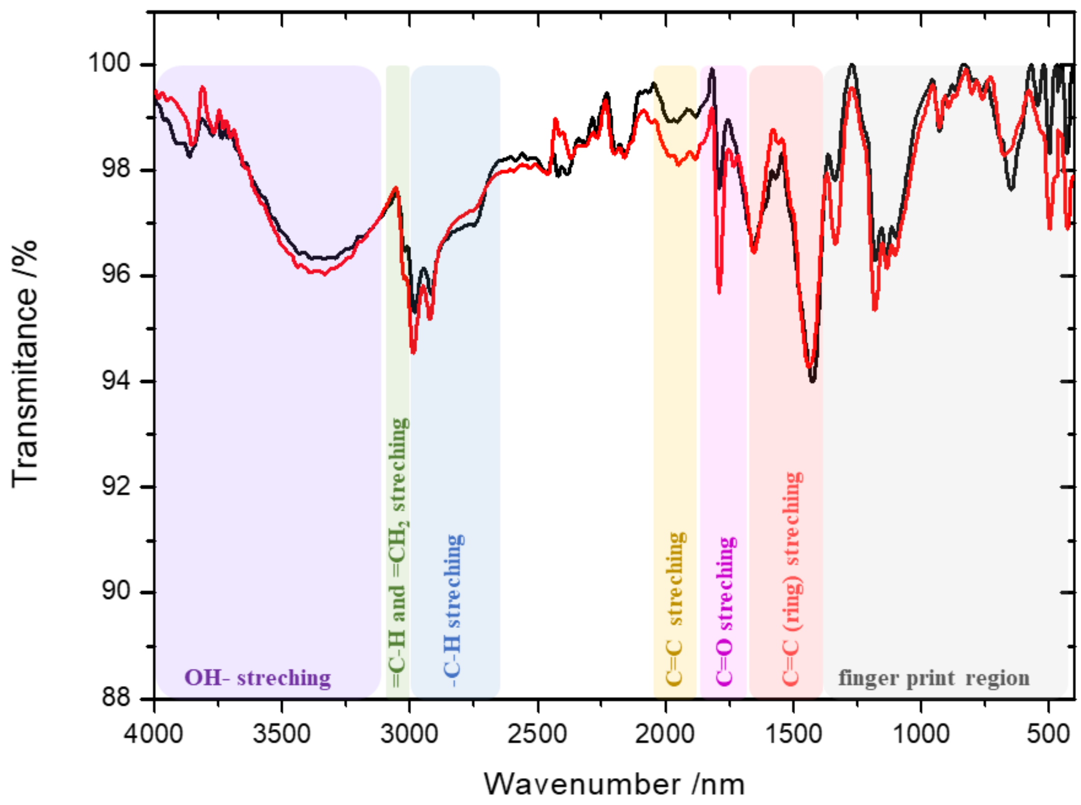

Figure S12. FTIR comparison for Ag NPs synthesized in the presence of the T_{hydro} (black line) and the T_{aquo} (red line) extracts.

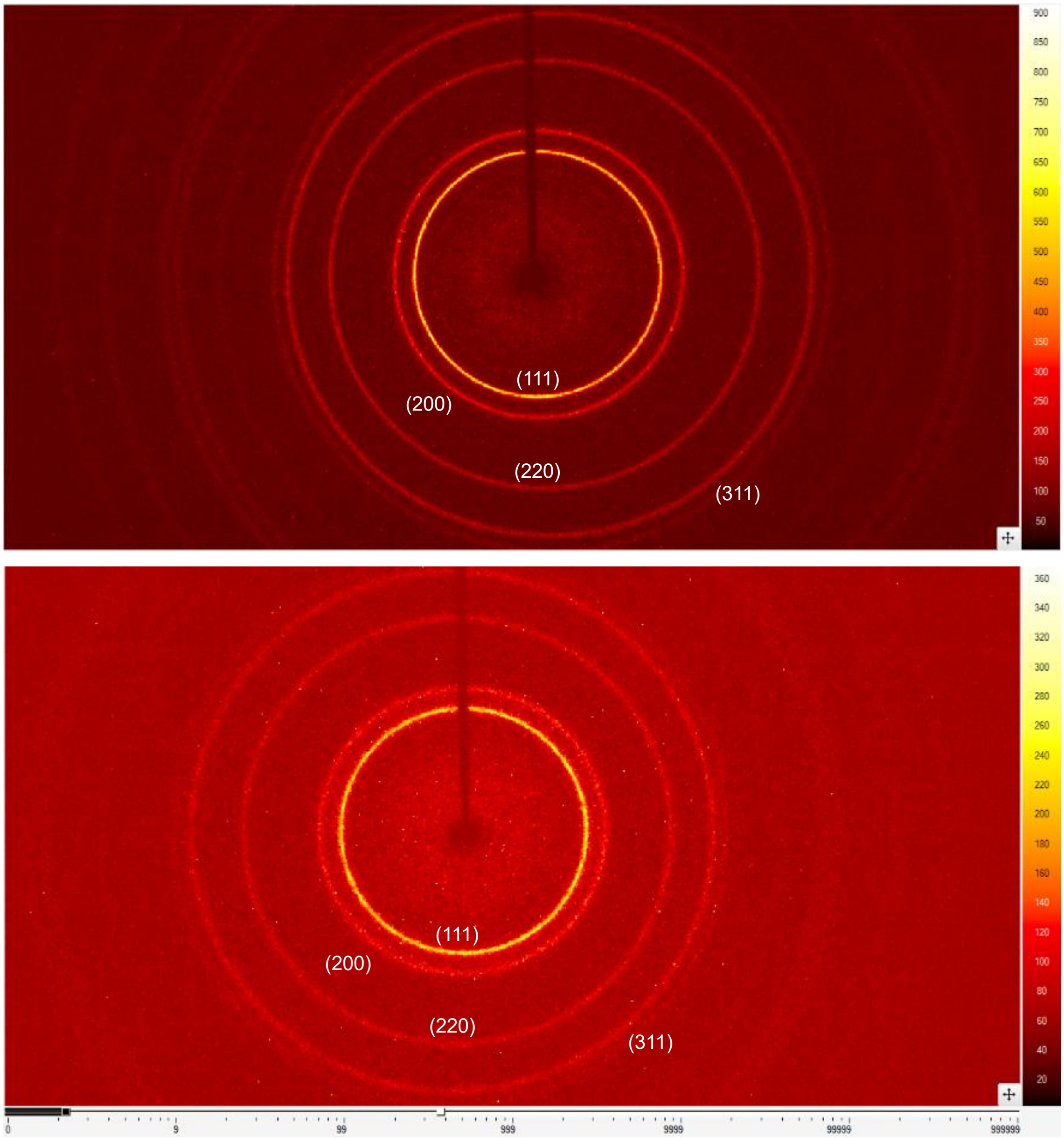

Figure S13. Selected area electron diffraction (SAED) patterns of biosynthesized silver nanoparticles with T_{aquo} (top) and T_{hydo} (bottom).

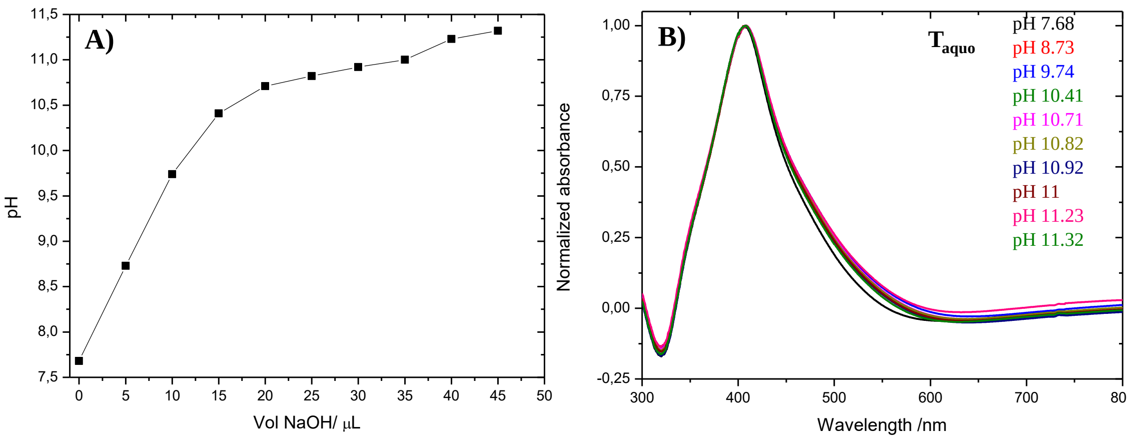

Figure S14. A) Evolution of the pH in a colloidal dispersion containing Ag NPs synthesized with the T_{aquo} extract after the addition of different aliquots of NaOH 0.1 M. B) UV-vis spectra for the Ag NPs dispersion at each pH value.

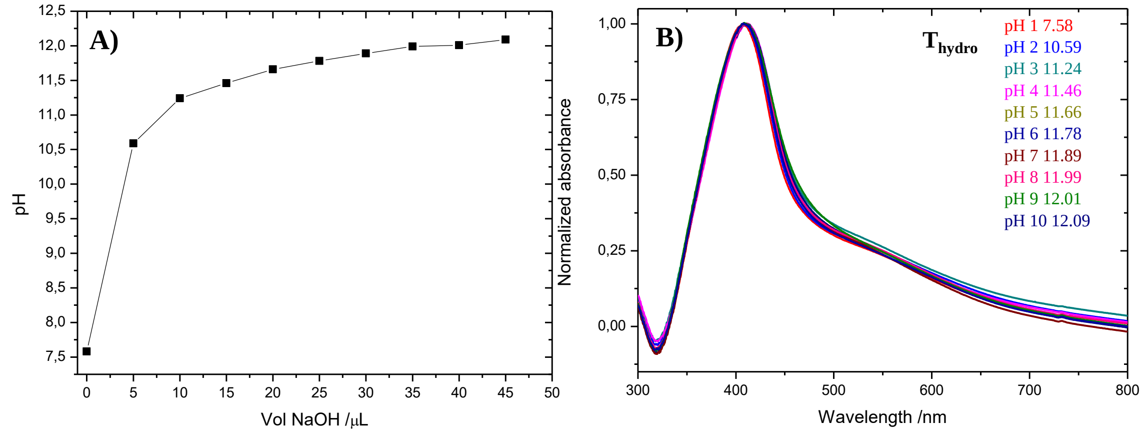

Figure S15. A) Evolution of the pH in a colloidal dispersion containing Ag NPs synthesized with the T_{hydro} extract after the addition of different aliquots of NaOH 0.1 M. B) UV-vis spectra for the Ag NPs dispersion at each pH value.

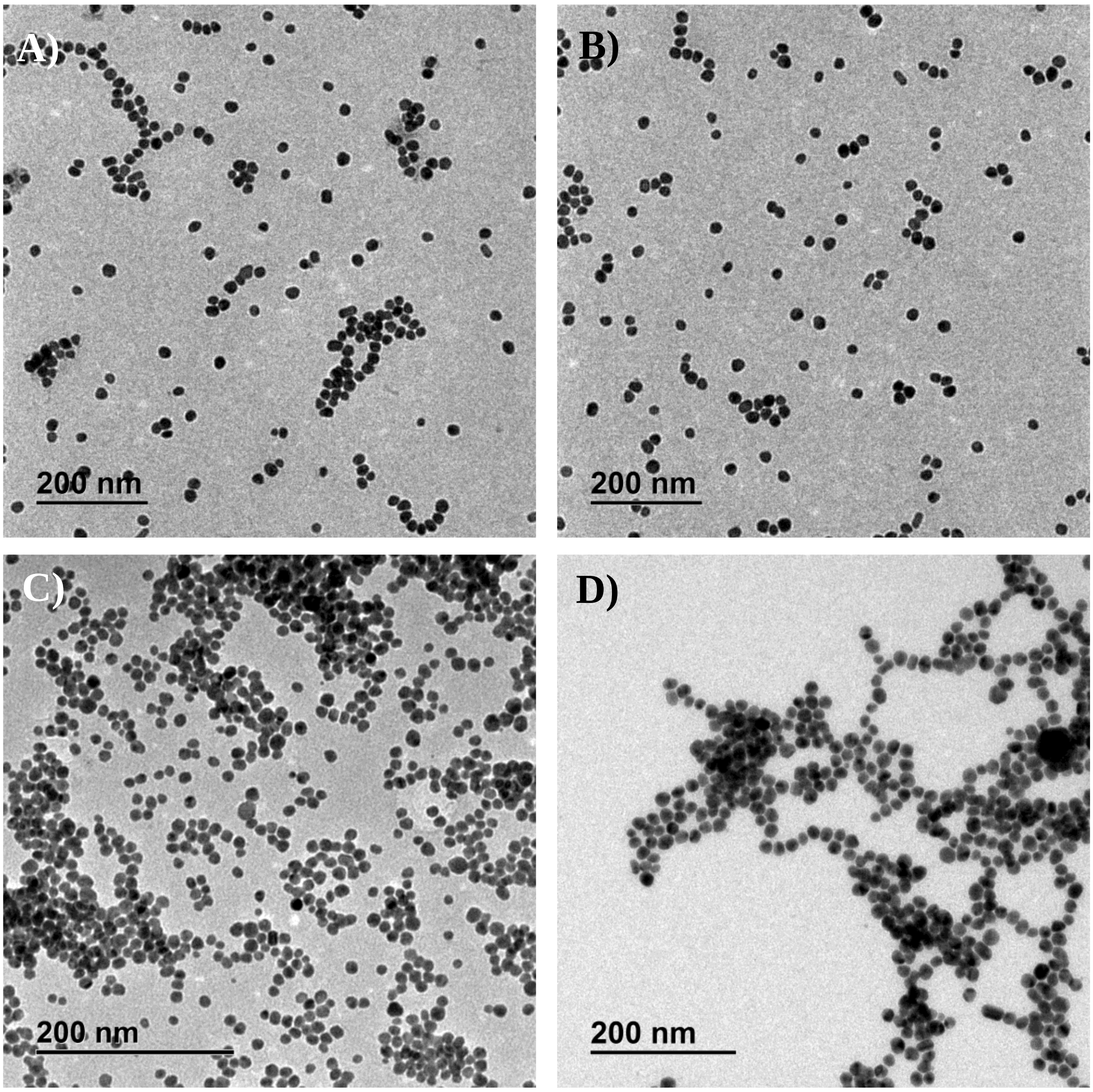

Figure S16. Representative TEM images for Ag NPs obtained with the T_{aquo} extract and after the addition different amounts of NaOH. The final pH values are A) 7.68, B)10.41, C) 10.92 and D) 11.32.

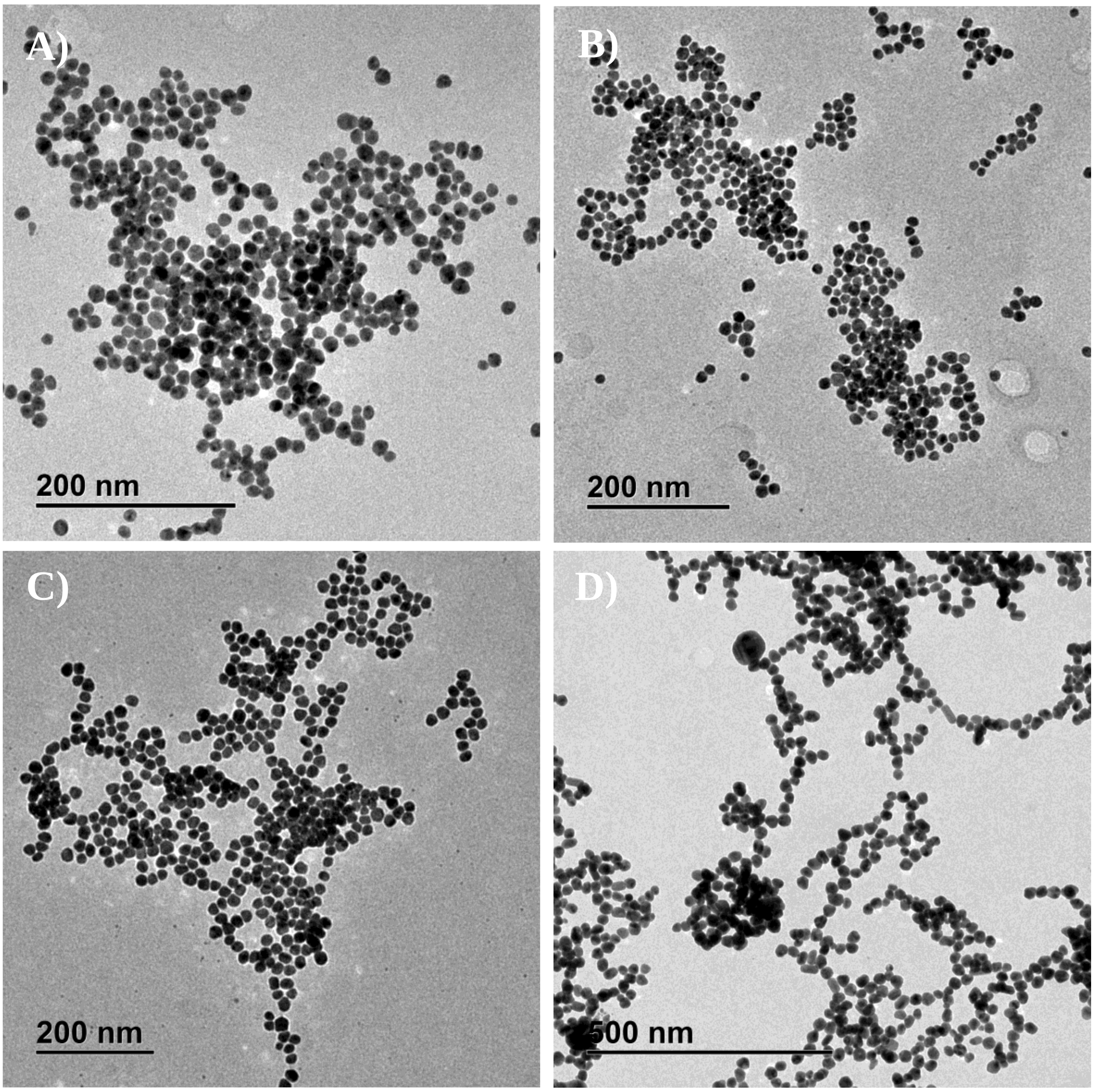

Figure S17. Representative TEM images for Ag NPs obtained with the T_{hydro} extract and after the addition different amounts of NaOH. The final pH values are A) 7.58, B) 10.59, C) 11.46 and D) 11.99.

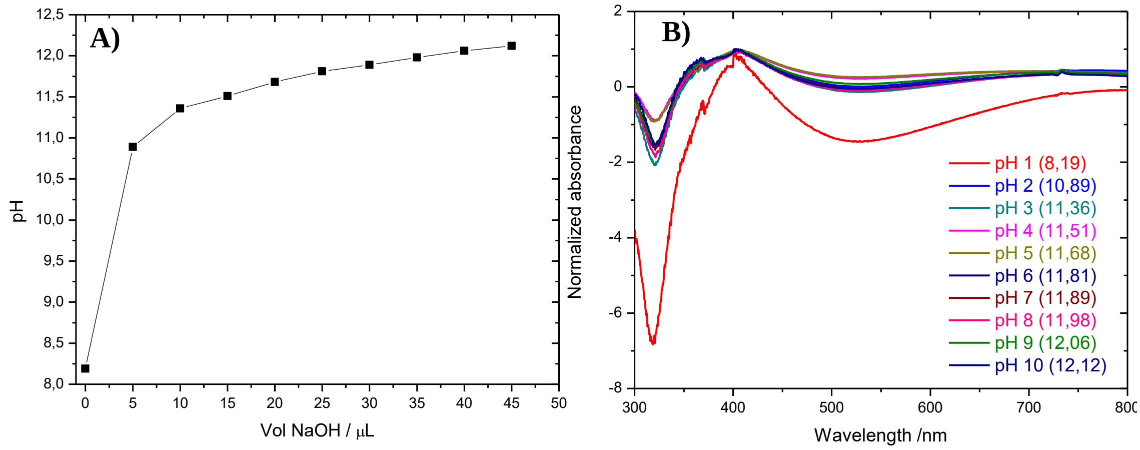

Figure S18. A) Evolution of the pH of a colloidal dispersion containing Ag NPs synthesized using $NaBH_4$ after the addition of different amounts of NaOH 0.1 M. B) UV-vis spectra for the Ag NPs dispersion at each pH value.

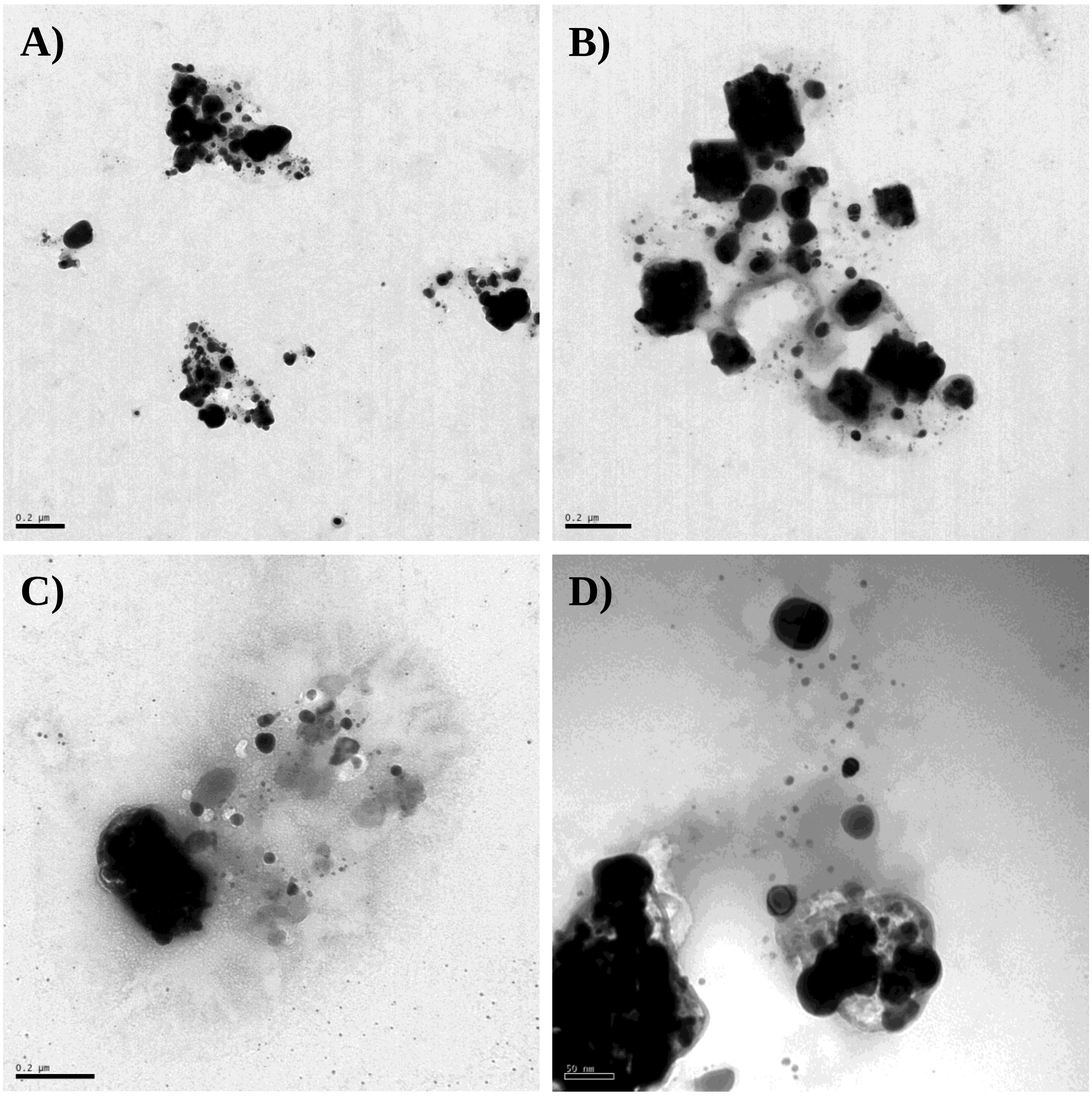

Figure S19. Representative TEM images for Ag NPs obtained using $NaBH_4$ and after the addition different amounts of NaOH. The final pH values are A) 8.19, B) 10.89, C) 11.51 and D) 11.98.

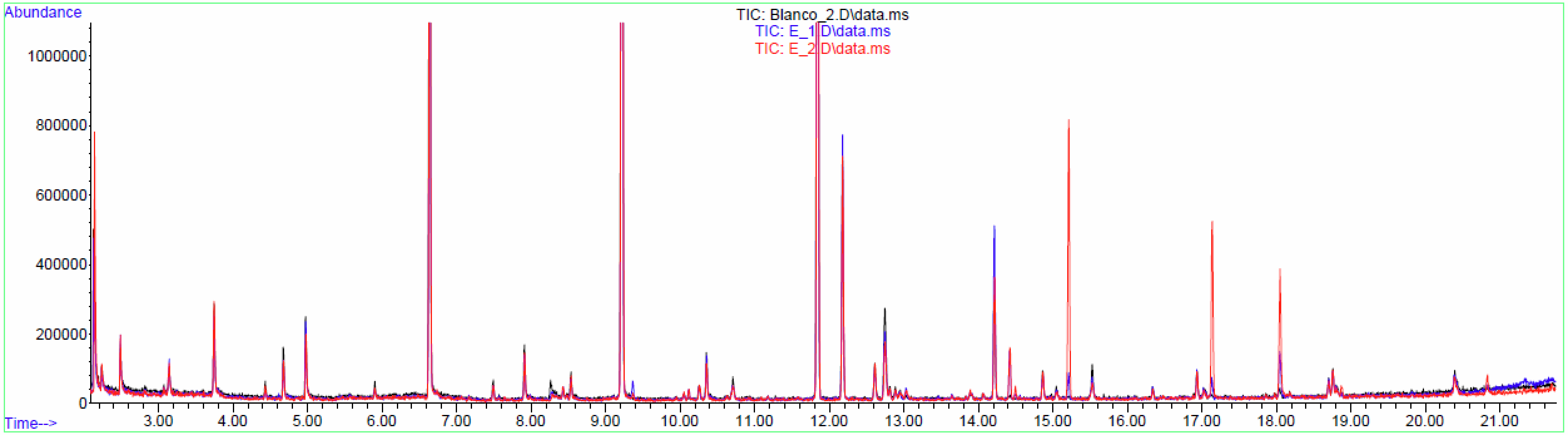

Figure S20. HS-SPME-GC-MS chromatographic profile of T_{hydro} (E_1 in blue line) and Ta_{quo} (E_2 in red line) Ag NPs together with a blank with no Ag NPs (Blanco_2 in black line) using DVB/CAR/PDMS fibers.

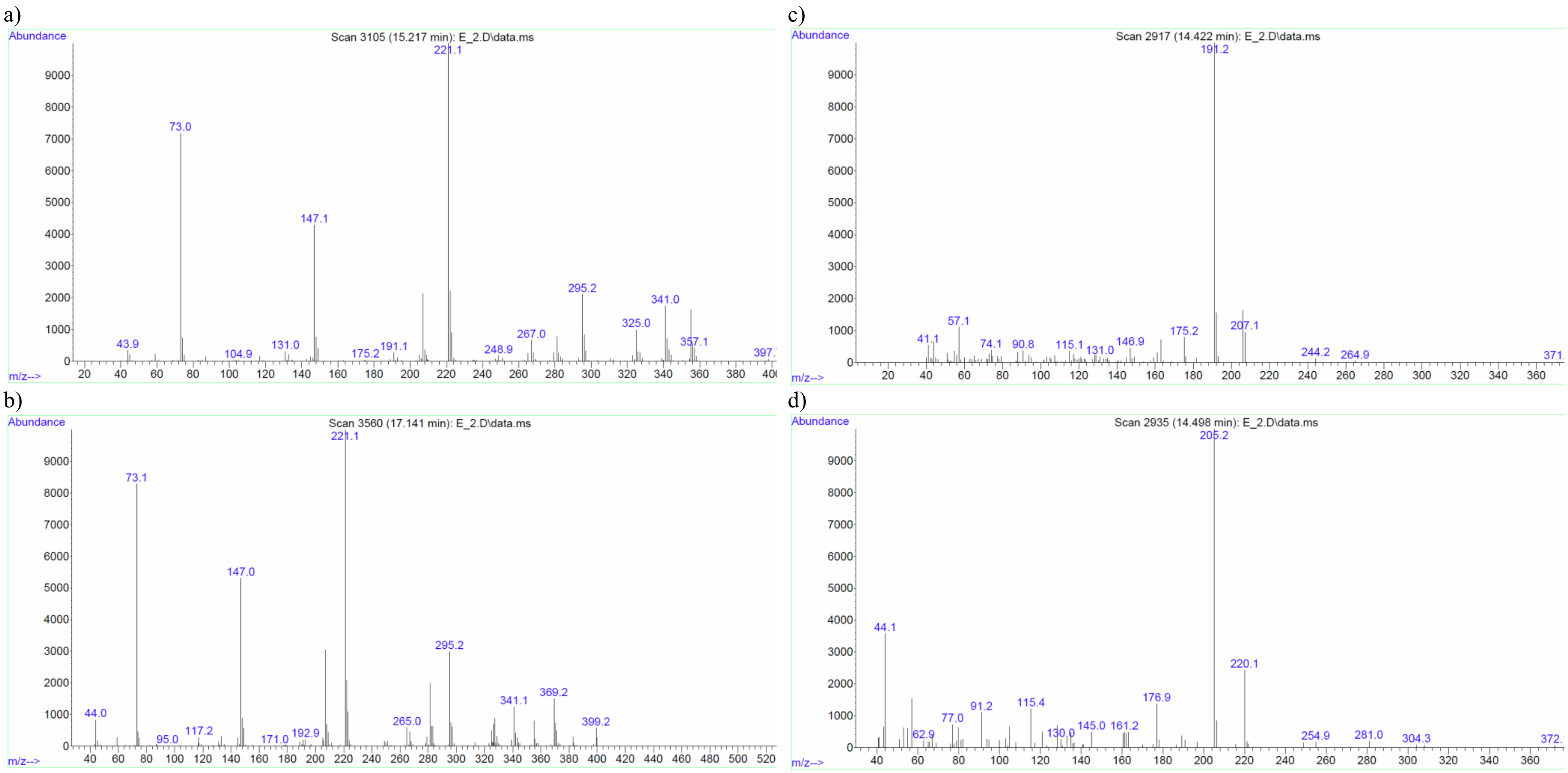

Figure S21. ESI–MS (ESI+) spectrum of peaks at retention times a) 15.2 min, b) 17.1 min, c) 14.4 min, and d) 14.5 min.
